# miR-10a rejuvenates aged human mesenchymal stem cells and improves heart function after myocardial infarction through KLF4

**DOI:** 10.1186/s13287-018-0895-0

**Published:** 2018-05-30

**Authors:** Jun Dong, Zhenhui Zhang, Hongshen Huang, Pei Mo, Chuanfan Cheng, Jianwei Liu, Weizhao Huang, Chaowei Tian, Chongyu Zhang, Jiao Li

**Affiliations:** 1 Guangzhou Institute of Cardiovascular Disease, Department of Cardiology, The Second Affiliated Hospital of Guangzhou Medical University, Guangzhou, 510260 China; 2 Department of Oncology, The Second Affiliated Hospital of Guangzhou Medical University, Guangzhou, China; 3 Department of Intensive Care Unit, The Second Affiliated Hospital of Guangzhou Medical University, Guangzhou, China; 4 Toronto General Research Institute, University Health Network, Toronto, Canada

**Keywords:** Aging, MicroRNA, Rejuvenation, Myocardial infarction

## Abstract

**Background:**

Aging is one of the key factors that regulate the function of human bone marrow mesenchymal stem cells (hBM-MSCs) and related changes in microRNA (miRNA) expression. However, data reported on aging-related miRNA changes in hBM-MSCs are limited.

**Methods:**

We demonstrated previously that miR-10a is significantly decreased in aged hBM-MSCs and restoration of the miR-10a level attenuated cell senescence and increased the differentiation capacity of aged hBM-MSCs by repressing Krüpple-like factor 4 (KLF4). In the present study, miR-10a was overexpressed or KLF4 was downregulated in old hBM-MSCs by lentiviral transduction. The hypoxia-induced apoptosis, cell survival, and cell paracrine function of aged hBM-MSCs were investigated in vitro. In vivo, miR-10a-overexpressed or KLF4-downregulated old hBM-MSCs were implanted into infarcted mouse hearts after myocardial infarction (MI). The mouse cardiac function of cardiac angiogenesis was measured and cell survival of aged hBM-MSCs was investigated.

**Results:**

Through lentivirus-mediated upregulation of miR-10a and downregulation of KLF4 in aged hBM-MSCs in vitro, we revealed that miR-10a decreased hypoxia-induced cell apoptosis and increased cell survival of aged hBM-MSCs by repressing the KLF4–BAX/BCL2 pathway. In vivo, transplantation of miR-10a-overexpressed aged hBM-MSCs promoted implanted stem cell survival and improved cardiac function after MI. Mechanistic studies revealed that overexpression of miR-10a in aged hBM-MSCs activated Akt and stimulated the expression of angiogenic factors, thus increasing angiogenesis in ischemic mouse hearts.

**Conclusions:**

miR-10a rejuvenated aged hBM-MSCs which improved angiogenesis and cardiac function in injured mouse hearts.

**Electronic supplementary material:**

The online version of this article (10.1186/s13287-018-0895-0) contains supplementary material, which is available to authorized users.

## Background

Previous studies proved that bone marrow (BM) stem cells play an important role in improving heart function and delaying cardiac remodeling [1, 2] after ischemic damage through reducing fibrosis and increasing angiogenesis. However, preclinical animal research does not necessarily translate into clinical results. Discrepancy in the effects of stem cell therapy [3, 4] may be due to the age of the donor [5]. The age of stem cell donors might significantly impact the recipient’s endogenous responses [6]. Our group found that the proliferation and differentiation ability of aged human bone marrow mesenchymal stem cells (hBM-MSCs) was decreased whereas cell senescence was increased [7]. Effective methods to rejuvenate aged hBM-MSCs to improve their regenerative capability may be required to maximize the beneficial effects of stem cell therapy.

Previous evidence has suggested that miRNAs regulate cell growth, differentiation, replication, survival, and senescence [8, 9]. We have reported that miR-26 attenuated myocardial hypertrophy by repressing expression of the GSK3b signaling pathway [10]. miR-30 decreased autophagy-mediated angiotensin II-related myocardial hypertrophy [11]. Furthermore, studies have shown that the aging process may change the expression pattern of miRNAs. This aging-related change in miRNA expression may play an important role in the regulation of cellular function [12]. Our previous study found that miR-196a, miR-486-5p, miR-664-star, and miR-378-star were significantly increased whereas miR-10a, miR-708, and miR-3197 were decreased in old hBM-MSCs. We also found that miR-10a increased differentiation and decrease senescence in old hBM-MSCs through the target gene Krüpple-like factor 4 (KLF4) [7]. However, other studies have suggested that miR-10a may have additional effects other than regulating cell senescence and differentiation. Indeed, some studies have shown that miR-10a and KLF4 regulate cell apoptosis in other cell types [13, 14]. We speculated that miR10a may also have additional effects related to cellular apoptosis and survival to regulate aged hBM-MSC function.

The effect of miR-10a on cellular apoptosis has been reported recently. Li et al. [13] found that miR-10a and miR-10b were significantly increased in CD34^+^ marrow cells from 28 patients with myelodysplastic syndrome compared with healthy donors. They also found that downregulation of miR10a/10b in clonal cells interfered with cell proliferation and enhanced cell apoptosis by activating the NF-κB-dependent p53 pathway [13]. Another study has also shown that miR-10a inhibited KLF4 and RB1-inducible coiled-coil 1(RB1CC1) regulated cell apoptosis in acute myeloid leukemia (AML) [14]. On the other hand, KLF4, one of the miR-10a target genes, has been reported to play a role in regulating cell apoptosis. In human malignant neuroblastoma SK-N-DZ and IMR-32 cell lines, upregulation of KLF4 inhibited cell growth and increased cell apoptosis by activating caspase-3 [15]. In leukemia cells, KLF4 upregulated Bax and downregulated Bcl-2 to induce cell apoptosis, potentially through binding to the sites which correspond to the promoters of Bcl-2 and Bax [16]. Furthermore, upregulation of KLF4 in the B-lymphoma cell line induced cell apoptosis through activation of BAK1 [17]. All of this evidence led us to postulate that miR-10a may mitigate cellular apoptosis of aged hBM-MSCs through its target gene KLF4.

The efficacy of stem cell therapy for ischemic diseases was compromised by the poor survival of MSCs [18]. In the current study, we investigated whether upregulation of miR-10a or downregulation of KLF4 can rejuvenate aged hBM-MSCs and improve aged hBM-MSC survival when transplanted into ischemic mouse hearts. In this regard, hBM-MSCs were harvested from young and old patients undergoing cardiac surgery. Overexpression or inhibition of miR-10a or KLF4 in hBM-MSCs was achieved through lentiviral transduction. The effects of miR-10a on hBM-MSC apoptosis, survival, and paracrine function were investigated in vitro. In vivo, miR-10a-overexpressed hBM-MSCs were implanted into the border region of mouse hearts following myocardial infarction (MI), and cardiac function and related biological changes were investigated. The molecular mechanisms of miR-10a on hBM-MSC function were further investigated.

## Methods

### Human bone marrow mesenchymal stem cell isolation, culture, and identification

This study was approved by the Research Ethics Committee of The Second Affiliated Hospital of Guangzhou Medical University. Human bone marrow (BM) was collected during cardiac valve surgery at The Second Affiliated Hospital of Guangzhou Medical University. After anesthesia induction but before completion of the sternotomy, 10 ml of sternal marrow was aspirated from the patients. Young human bone marrow mesenchymal stem cells (hBM-MSCs) were obtained from patients 18–30 years old, and old hBM-MSCs from patients 65–80 years old. All patients were free of other pathological conditions.

The hBM-MSCs were cultured as described previously [7]. Briefly, after centrifugation through a Ficoll gradient (1.077 g/ml density; GE Healthcare, Kretztechnik, Zipf, Austria), cells were separated and the mononuclear cell fraction was plated. After 48 h, nonadherent cells were removed by changing the culture medium. The adherent cells were passaged when confluency reached 80%. hBM-MSCs were characterized by flow cytometry after staining with antibodies against CD29, CD31, CD34, CD44, CD45, and CD166 (MultiSciences Biotech Co., Shanghai, China) as described previously.

### Viral vector construction and transduction

Lentiviral constructs for overexpression of miR-10a (O-10a), KLF4 (O-KLF4), or inhibition of miR-10a and KLF4 (O-anti10a and O-antiKLF4) in hBM-MSCs were purchased from GenePharma as reported previously [7]. The Lenti-miR-10a sequence was TACCCTGTAGATCCGAATTTGTG. The Lenti-anti-10a sequence was CACAAATTCGGATCTACAGGGTA. The Lenti-anti-KLF4 sequence was GCCACCCACACTTGTGATTAC. The Lenti-control sequence was TTCTCCGAACGTGTCACGT. To construct LV-KLF4, the entire CDS region of KLF4 (NCBI reference sequence: NM_004235.4) was subcloned into the lentiviral vector. All of the lentiviral constructs expressed GFP. Viral solution was diluted with serum-free medium and polybrene (5 mg/ml) was added to the culture medium to transduce hBM-MSCs for 72 h.

### Cell survival and apoptosis evaluation

Young (Y) and old hBM-MSCs (O) were cultured for 72 h under hypoxia conditions (0.1% O_2_). Cell survival was determined using the Cell Counting Kit-8 (CCK8; Dojindo, Kumamoto, Japan) according to the manufacturer’s instructions. In brief, cells were seeded into 96-well plates at a density of 1 × 10^4^ cells/well. After incubation, 5 μl of CCK-8 reagent was added to each well. The absorbance was measured at 450 nm after 2 h of incubation at 37 °C.

Terminal deoxynucleotidyl transferase dUTP nick end labeling (TUNEL; Roche, Laval, QC, Canada) was carried out according to the manufacturer’s instructions. The number of TUNEL^+^ cells in five randomly selected high-power fields per dish was determined and averaged with a Nikon Eclipase Ti fluorescent microscope. Cell apoptosis was expressed as the percentage of total cells (DAPI^+^).

### Real-time reverse transcription-polymerase chain reaction

Total RNA was isolated with TRIzol reagent (Invitrogen, Grand Island, NY, USA) and cDNA was synthesized using Moloney murine leukemia virus reverse transcriptase and random primer. Real-time polymerase chain reaction was conducted using SensiFAST SYBR Green PCR Master Mix (Bioline USA Inc., Taunton, MA, USA) with the following parameters: 95 °C for 2 min, 95°C for 5 s, and 60 °C for 30 s for 40 cycles. The oligonucleotide primer sequences are presented in Additional file 1: Table S1.

### Myocardial infarction and cardiac function measurement

The animal protocol was approved by Research Ethics Committee of The Second Affiliated Hospital of Guangzhou Medical University (2014013). Coronary occlusion was performed as described previously [19]. Briefly, C57BL/6 mice were intubated and ventilated with 2% isoflurane. Through a left thoracotomy, the left anterior descending coronary artery was ligated. Cardiac function was measured with echocardiography at different time points before and after MI as indicated in Results. At the study end, the scar area and thickness were measured by planimetry. The infarct area was defined as the entire area of the left ventricle that contained a scar in myocardial sections stained with Masson’s trichrome. Three days prior to MI and cell transplantation, mice were given daily doses of cyclosporine A (5 mg/kg) to induce the immunosuppression duration of the experiments.

### Immunofluorescent staining

Hearts were fixed in 2% paraformaldehyde (PFA) for 24 h after being well perfused with PFA and were then stored in 0.5 M sucrose at 4 °C overnight. Hearts were then embedded with OCT, and 5-μm-thick frozen sections were prepared. Slides were incubated with one of the following primary antibodies: Alexa488-conjugated anti-GFP (catalog number A21311, 1:400; Invitrogen) or anti-α-SMA (catalog number A-2547, 1:400; Sigma), at room temperature for 2 h. Incubation with the respective Alexa488 or Alexa568-conjugated secondary antibodies (all 1:400; Invitrogen) or Isolectin B4 (catalog number I21412, 1:100; ThermoFisher) was carried out at room temperature with light protection for 1 h. The nuclei were identified with DAPI.

### Western blotting

For western blot analysis, 50 μg of lysate was fractionated through a 4% stacking and 10% running SDS-PAGE gel, and the fractionated proteins were transferred to a PVDF membrane. Blots were blocked for 1 h at room temperature with blocking buffer. The antibodies (VEGF, SDF, phosphor (ser473)-AKT, and total AKT, all 1:1000) reacted with the blots overnight at 4 °C. After washing (3 × 5 min in 1 × TBS–0.1% Tween 20), the blots were incubated with horseradish peroxidase-conjugated secondary antibody at 1:2000 dilution for 1 h at room temperature. Visualization was performed with enhanced chemiluminescence. For quantification, densitometry of the target bands was divided by the corresponding densitometry of the GAPDH (catalog number mab374, 1:5000; Millipore) band using ImageJ software. To inhibit Akt phosphorylation in the miR-10a-overexpressed old hBM-MSCs, 50 μM Akt Inhibitor VI (catalog number 124013; Merck) was used to treat the cells for 72 h under hypoxia conditions following the manufacturer’s instructions. The inhibited expression of P-AKT was conferred by western blotting.

### Enzyme-linked immunosorbent assay

hBM-MSCs in different groups were cultured in serum-free DMEM medium under hypoxia conditions for 72 h. The supernatant and cell lysate were collected and the protein concentration was determined using a Bio-Rad DC protein assay kit. The level of VEGF (catalog number DVE00; R&D Systems) and SDF (catalog number DSA00; R&D Systems) were determined using an enzyme-linked immunosorbent assay (ELISA) following the manufacturer’s instructions.

### Caspase activity assay

A caspase fluorescent assay kit specific for caspase-3 (Biovision, Mountain View, CA, USA) was used to detect caspase activation by measuring the cleavage of a synthetic fluorescent substrate. In brief, cell lysates were prepared with the lysis buffer provided by the assay kit and centrifuged at 10,000 × *g* for 1 min, and the supernatants were collected. With bovine serum albumin as the standard for protein content, equal amounts of protein were reacted with the synthetic fluorescent substrates at 37 °C for 1.5 h, and absorbance at 405 nm was read on a microplate reader. The fold-increase in caspase-3 activity versus control was determined.

### Statistical analysis

All values are expressed as mean ± SD. Analyses were performed using GraphPad InStat software (version 6). Student’s *t* test was used for two-group comparisons. Comparisons of parameters among three or more groups were analyzed using one-way analysis of variance (ANOVA) followed by Tukey for single-factor variables or two-way ANOVA for two-factor variables with repeated measures over time, followed by Bonferroni post-hoc tests. Differences were considered statistically significant at *p* < 0.05.

## Results

### Cell apoptosis was increased in old hBM-MSCs under hypoxia conditions

**Fig. 1:**
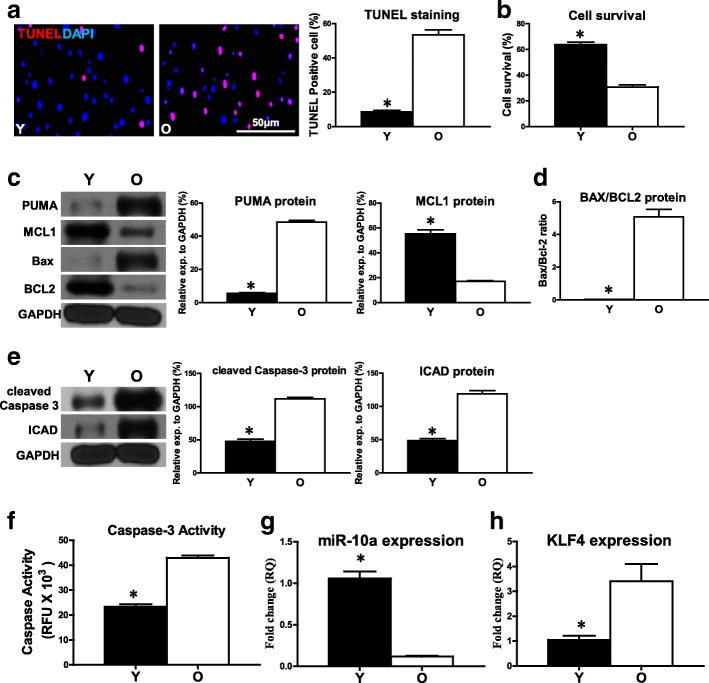
Cell apoptosis increased in old hBM-MSCs under hypoxia conditions. Young (Y) and old (O) hBM-MSCs cultured for 72 h under hypoxia conditions. **a** Cell apoptosis assayed by TUNEL staining. Percentage of apoptotic cells (TUNEL^+^) quantified in Y and O hBM-MSCs. **b** Cell survival evaluated in Y and O hBM-MSCs **c** Protein expression of MCL1 and PUMA evaluated by western blot analysis in Y and O hBM-MSCs. **d** Ratio of Bax/BCL2 quantified in Y and O hBM-MSCs. **e** Protein expression of cleaved caspase-3 and inhibitor of caspase-activated DNase (ICAD) assayed in Y and O hBM-MSCs. **f** Caspase-3 activity measured in Y and O hBM-MSCs. Expression of (**g)** miR-10a and (**h**) KLF4 compared in Y and O hBM-MSCs. *n* = 6/group. Mean ± SD. *P < 0.05. DAPI 4′,6-diamidino-2-phenylindole, KLF4 Krüpple-like factor 4, TUNEL terminal deoxynucleotidyl transferase dUTP nick end labeling, RQ relative quantity, RFU relative fluorescence units

Young (Y) and old (O) hBM-MSCs were cultured for 72 h under hypoxia conditions, followed by comparison of cell survival and apoptosis. The percentage of apoptotic cells (TUNEL^+^) was significantly higher in the O group compared with the Y group of hBM-MSCs (Fig. 1a). In agreement, cell survival was decreased in O hBM-MSCs compared with Y hBM-MSCs by CCK-8 assay (Fig. 1b). The proapoptotic mRNA expression of BAX and PUMA was significantly higher in O hBM-MSCs compared with Y hBM-MSCs (Additional file 2: Figure S1). On the contrary, the antiapoptotic mRNA expression of BCL2 and MCL1 (BCL2 family apoptosis regulator) was significantly lower in O hBM-MSCs compared with Y hBM-MSCs (Additional file 2: Figure S1). The proapoptotic protein expression of PUMA was also significantly higher whereas the antiapoptotic protein expression of MCL1 was significantly lower in O hBM-MSCs compared with Y hBM-MSCs respectively (Fig. 1c). The ratio of BAX/BCL2 protein was increased in O hBM-MSCs compared with Y hBM-MSCs (Fig. 1d). The protein expression of cleaved caspase-3 and inhibitor of caspase-activated DNase (ICAD) was also increased in O hBM-MSCs compared with Y hBM-MSCs (Fig. 1e). Furthermore, caspase-3 activity was significantly higher in O hBM-MSCs than in Y hBM-MSCs (Fig. 1f). The expression of miR-10a was significantly decreased in O hBM-MSCs compared with Y hBM-MSCs (Fig. 1g). To the contrary, the expression of KLF4, which was one of the targets of miR-10a, was significantly increased in O hBM-MSCs compared with Y hBM-MSCs (Fig. 1h). All of these data implied the possible link between the downregulation of miR-10a and the increased O hBM-MSC apoptosis.

### Upregulation of miR-10a in old hBM-MSCs decreased hypoxia-induced apoptosis and increased cell survival

**Fig. 2:**
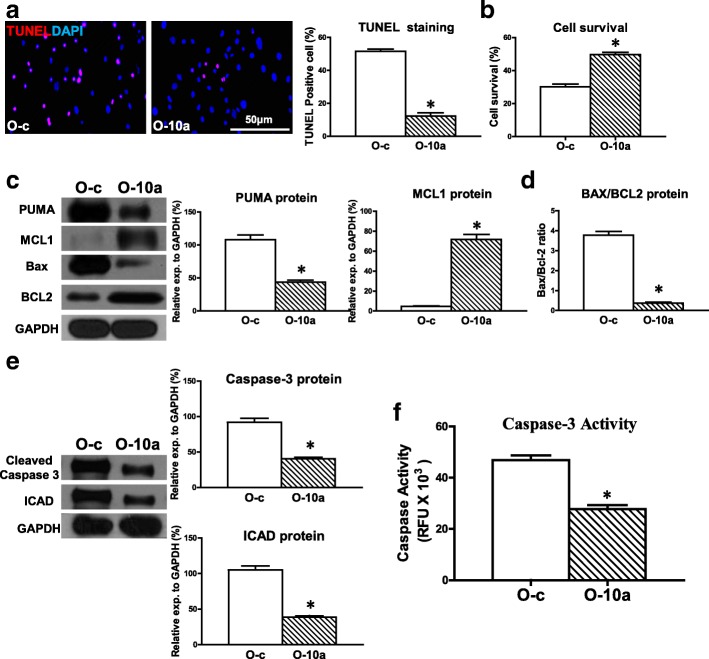
Overexpression of miR-10a in old hBM-MSCs decreased hypoxia-induced apoptosis and increased cell survival. miR-10a transduced into old hBM-MSCs by lentiviral vector (O-10a). Control vector-transduced old hBM-MSCs (O-c) served as control. Cells cultured for 72 h under hypoxia conditions. **a** Cell apoptosis assayed by TUNEL staining. Percentage of apoptotic cells (TUNEL^+^) quantified in O-c and O-10a hBM-MSCs. **b** Cell survival evaluated in O-c and O-10a hBM-MSCs. **c** Protein expression of MCL1 and PUMA evaluated by western blot analysis in O-c and O-10a hBM-MSCs. **d** Ratio of Bax/BCL2 quantified in O-c and O-10a hBM-MSCs. **e** Protein expression of cleaved caspase-3 and inhibitor of caspase-activated DNase (ICAD) assayed in O-c and O-10a hBM-MSCs. **f** Caspase-3 activity measured in O-c and O-10a hBM-MSCs. *n* = 6/group. Mean ± SD. *P < 0.05. DAPI 4′,6-diamidino-2-phenylindole, TUNEL terminal deoxynucleotidyl transferase dUTP nick end labeling, RFU relative fluorescence units

Next, to further test whether miR-10a was related to O hBM-MSC apoptosis, miR-10a was overexpressed in O hBM-MSCs (Additional file 3: Figure S2A) and cellular apoptosis was evaluated. The percentage of apoptotic cells (TUNEL^+^) was decreased in miR-10a-upregulated O hBM-MSCs (O-10a) compared with the control vector-transduced O hBM-MSCs (O-c) that were cultured for 72 h under hypoxia conditions (Fig. 2a). In agreement, cell survival was increased in the O-10a group compared with the O-c group (Fig. 2b). The proapoptotic mRNA expression of BAX and PUMA was decrease in the O-10a group compared with the O-c group (Additional file 4: Figure S3). On the contrary, the antiapoptotic mRNA expression of BCL2 and MCL1 was increased in the O-10a group compared with the O-c group (Additional file 4: Figure S3). The proapoptotic protein expression of PUMA was decreased whereas the antiapoptotic protein expression of MCL1 was increased in the O-10a group compared with the O-c group respectively (Fig. 2c). The ratio of BAX/BCL2 protein in the O-10a group was decreased compared with the O-c group (Fig. 2d). The protein expression of cleaved caspase-3 and ICAD was decreased in the O-10a group compared with the O-c group (Fig. 2e). Furthermore, caspase-3 activity was significantly lower in the O-10a group compared with the O-c group (Fig. 2f). These findings suggested that restoring the miR-10a level reduced hypoxia-induced apoptosis in O hBM-MSCs. We also examined the effects of miR-10a on Y hBM-MSCs and found that cell apoptosis was decreased (Additional file 5: Figure S4A) and cell survival (Additional file 5: Figure S4B) was increased in miR-10a-upregulated Y hBM-MSCs (Y-10a) compared with the control vector-transduced Y hBM-MSCs (Y-c). The effects of miR-10a on Y hBM-MSC apoptosis and survival followed the same trend as in O hBM-MSCs but at a much lower magnitude.

### Downregulation of KLF4 expression in old hBM-MSCs decreased hypoxia-induced apoptosis and increased cell survival

**Fig. 3:**
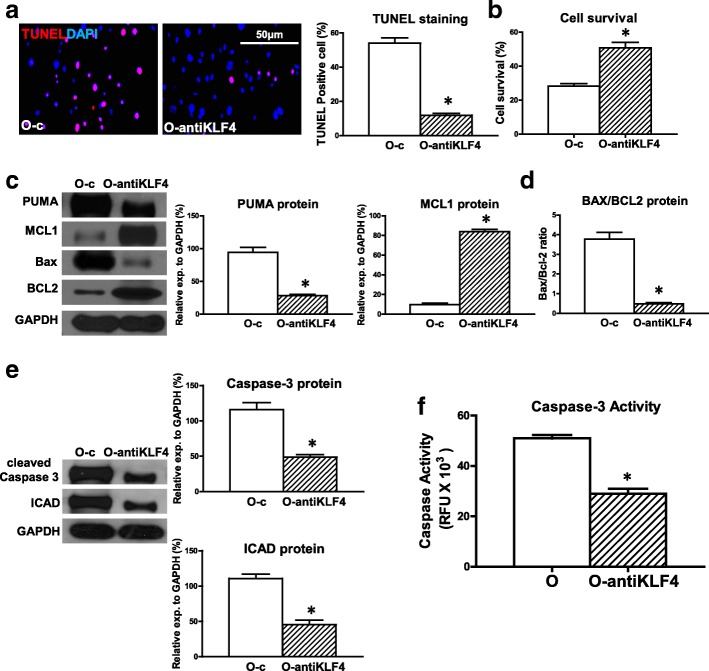
Downregulation of KLF4 in old hBM-MSCs decreased hypoxia-induced apoptosis and increased cell survival. KLF4 was effectively inhibited in old hBM-MSCs by lentiviral vector (O-antiKLF4). Control vector-transduced old hBM-MSCs (O-c) served as control. Cells cultured for 72 h under hypoxia conditions. **a** Cell apoptosis assayed by TUNEL staining. Percentage of apoptotic cells (TUNEL^+^) quantified in O-c and O-antiKLF4 hBM-MSCs. **b** Cell survival evaluated in O-c and O-antiKLF4 hBM-MSCs. **c** Protein expression of MCL1 and PUMA evaluated by western blot analysis in O-c and O-antiKLF4 hBM-MSCs. **d** Ratio of Bax/BCL2 quantified in O-c and O-antiKLF4 hBM-MSCs. **e** Protein expression of cleaved caspase-3 and inhibitor of caspase-activated DNase (ICAD) assayed in O-c and O-antiKLF4 hBM-MSCs. **f** Caspase-3 activity measured in O-c and O-antiKLF4 hBM-MSCs. *n* = 6/group. Mean ± SD. *P < 0.05. DAPI 4′,6-diamidino-2-phenylindole, KLF4 Krüpple-like factor 4, TUNEL terminal deoxynucleotidyl transferase dUTP nick end labeling, RFU relative fluorescence units

KLF4 is one of the targets of miR-10a and was upregulated in O hBM-MSCs. To determine whether KLF4 was involved in miR-10a-mediated cellular apoptosis, we effectively inhibited KLF4 in O hBM-MSCs (Additional file 3: Figure S2B) and evaluated cell apoptosis. The percentage of apoptotic cells (TUNEL^+^) was decreased in the KLF4-inhibited O hBM-MSCs (O-antiKLF4) compared with the control vector-transduced O hBM-MSCs (O-c) that were cultured for 72 h under hypoxia conditions (Fig. 3a). In agreement, cell survival was increased in the O-antiKLF4 group compared with the O-c group (Fig. 3b). The proapoptotic mRNA expression of BAX and PUMA was decreased in the O-antiKLF4 group compared with the O-c group (Additional file 6: Figure S5). On the contrary, the antiapoptotic mRNA expression of BCL2 and MCL1 was increased in the O-antiKLF4 group compared with the O-c group (Additional file 6: Figure S5). The proapoptotic protein expression of PUMA was decreased whereas the antiapoptotic protein expression of MCL1 was increased in the O-antiKLF4 group compared with the O-c group respectively (Fig. 3c). The ratio of BAX/BCL2 protein in the O-antiKLF4 group was decreased when compared with the O-c group (Fig. 3d). Moreover, the protein expression of cleaved caspase-3 and ICAD was decreased in the O-antiKLF4 group compared with the O-c group (Fig. 3e). Caspase-3 activity was significantly lower in the O-antiKLF4 group compared with the O-c group (Fig. 3f).

### Downregulation of miR-10a or overexpression of KLF4 in old hBM-MSCs increased hypoxia-induced apoptosis and decreased cell survival

**Fig. 4:**
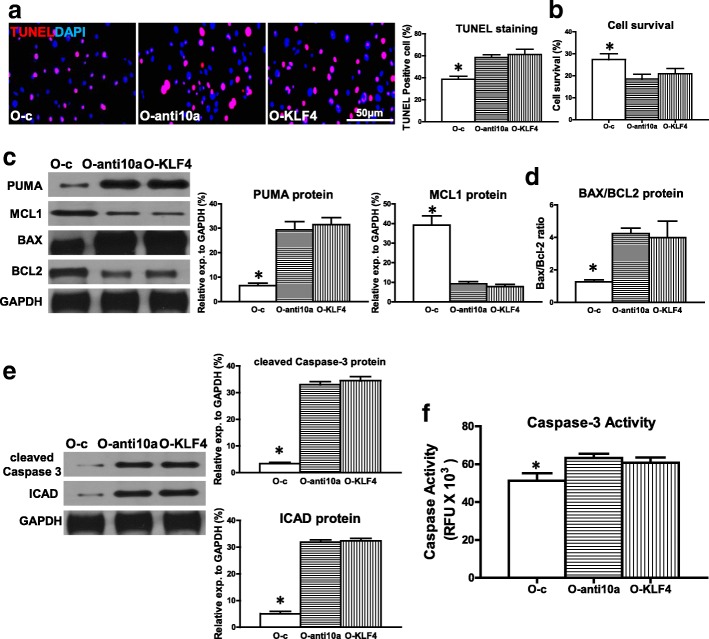
Downregulation of miR-10a or overexpression of KLF4 in old hBM-MSCs increased hypoxia-induced apoptosis and decreased cell survival. Lentiviral vectors used to transduce old hBM-MSCs to downregulate miR-10a expression (O-anti10a) or overexpress KLF4 (O-KLF4). Control vector-transduced old hBM-MSCs (O-c) served as control. Cells cultured for 72 h under hypoxia condition. **a** Cell apoptosis assayed by TUNEL staining. Percentage of apoptotic cells (TUNEL^+^) quantified in O-c, O-anti10a, and O-KLF4 hBM-MSCs. **b** Cell survival evaluated in O-c, O-anti10a, and O-KLF4 hBM-MSCs. **c** Protein expression of MCL1 and PUMA evaluated by western blot analysis in O-c, O-anti10a, and O-KLF4 hBM-MSCs. **d** Ratio of Bax/BCL2 quantified in O-c, O-anti10a, and O-KLF4 hBM-MSCs. **e** Protein expression of cleaved caspase-3 and inhibitor of caspase-activated DNase (ICAD) assayed in O-c, O-anti10a, and O-KLF4 hBM-MSCs. **f** Caspase-3 activity measured in O, O-anti10a, and O-KLF4 hBM-MSCs. *n* = 6/group. Mean ± SD. *P < 0.05. DAPI 4′,6-diamidino-2-phenylindole, KLF4 Krüpple-like factor 4, TUNEL terminal deoxynucleotidyl transferase dUTP nick end labeling, RFU relative fluorescence units

All of this evidence suggested that miR-10a, through suppression of KLF4, may rescue O hBM-MSCs from hypoxia-induced apoptosis. To test whether the reverse would post a detrimental effect, miR-10a was effectively inhibited in O hBM-MSCs (Additional file 3: Figure S2C) or KLF4 was overexpressed in O hBM-MSCs (Additional file 3: Figure S2D). The percentage of apoptotic cells (TUNEL^+^) was significantly higher in miR-10a-inhibited old hBM-MSCs (O-anti10a) and KLF4-overexpressed old hBM-MSCs (O-KLF4) compared with the control vector-transduced old hBM-MSCs (O-c) that were cultured for 72 h under hypoxia conditions (Fig. 4a). In agreement, cell survival was decreased in the O-anti10a and O-KLF4 groups compared with the O-c group (Fig. 4b). The proapoptotic mRNA expression of BAX and PUMA was significantly higher in the O-anti10a and O-KLF4 groups compared with the O-c group (Additional file 7: Figure S6). On the contrary, the antiapoptotic mRNA expression of BCL2 and MCL1was significantly lower in the O-anti10a and O-KLF4 groups compared with the O-c group (Additional file 7: Figure S6). The proapoptotic protein expression of PUMA was increased whereas the antiapoptotic protein expression of MCL1 was decreased in the O-anti10a and O-KLF4 groups compared with the O-c group respectively (Fig. 4c). The ratio of BAX/ BCL2 protein in the O-anti10a and O-KLF4 groups was increased when compared with the O-c group (Fig. 4d). The protein expression of cleaved caspase-3 and ICAD was increased in the O-anti10a and O-KLF4 groups compared with the O-c group (Fig. 4e). Caspase-3 activity was significantly higher in the O-anti10a and O-KLF4 groups compared with the O-c group (Fig. 4f).

### The antiapoptotic effect of miR-10a is attenuated by the restoration of KLF4

**Fig. 5:**
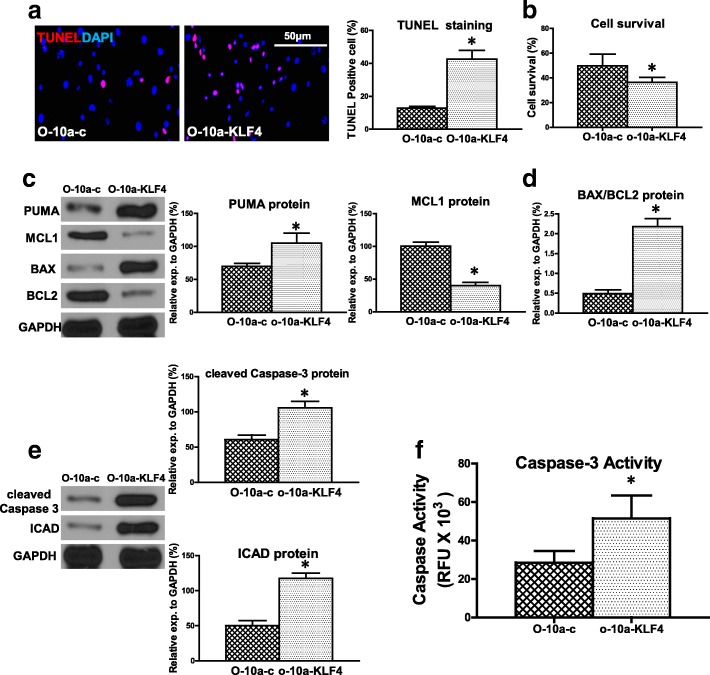
Antiapoptotic effect of miR-10a attenuated by restoration of KLF4. Lentivirus which carries KLF4 vector used to infect miR-10a-upregulated old hBM-MSCs (O-10a) to restore KLF4 expression (O-10a-KLF4). miR-10a-upregulated old hBM-MSCs (O-10a) also infected by the control lentivirus (O-10a-c). Cells cultured for 72 h under hypoxia conditions. **a** Cell apoptosis assayed by TUNEL staining. Percentage of apoptotic cells (TUNEL^+^) quantified in O-10a and O-10a-KLF4 hBM-MSCs. **b** Cell survival evaluated in O-10a and O-10a-KLF4 hBM-MSCs. **c** Protein expression of MCL1 and PUMA evaluated by western blot analysis in O-10a and O-10a-KLF4 hBM-MSCs. **d** Ratio of Bax/BCL2 quantified in O-10a and O-10a-KLF4 hBM-MSCs. **e** Protein expression of cleaved caspase-3 and inhibitor of caspase-activated DNase (ICAD) assayed in O-10a and O-10a-KLF4 hBM-MSCs. **f** Caspase-3 activity evaluated in O-10a and O-10a-KLF4 hBM-MSCs. *n* = 6/group. Mean ± SD. *P < 0.05. DAPI 4′,6-diamidino-2-phenylindole, KLF4 Krüpple-like factor 4, TUNEL terminal deoxynucleotidyl transferase dUTP nick end labeling, RFU relative fluorescence units

To further confirm that KLF4 is indeed the direct target of miR-10a in mediating hypoxia-induced O hBM-MSC apoptosis, KLF4 expression was restored by a rescue experiment (Additional file 3: Figure S2E). For this purpose, miR-10a-overexpressed O hBM-MSCs were transduced with a lentivirus which carried the KLF4 vector and the restoration of KLF4 expression was confirmed (O-10a-KLF4). After restoring the expression of KLF4, miR10a lost its antiapoptotic effect on O hBM-MSCs. Cell apoptosis was increased in the O-10a-KLF4 group compared with the O-10a group (Fig. 5a). In agreement, cell survival was decreased in the O-10a-KLF4 group compared with the O-10a group (Fig. 5b). The proapoptotic mRNA expression of BAX and PUMA was significantly higher in the O-10a-KLF4 group compared with O-10a hBM-MSCs. On the contrary, the antiapoptotic mRNA expression of BCL2 and MCL1 was significantly lower in the O-10a-KLF4 group compared with O-10a hBM-MSCs (Additional file 8: Figure S7). The proapoptotic protein expression of PUMA was increased whereas the antiapoptotic protein expression of MCL1 was decreased in the O-10a-KLF4 group compared with the O-10a group respectively (Fig. 5c). The ratio of BAX/ BCL2 protein was increased in the O-10a-KLF4 group compared with the O-10a group (Fig. 5d). The protein expression of active caspase-3 and ICAD was increased in the O-10a-KLF4 group compared with O-10a hBM-MSCs (Fig. 5e). Caspase-3 activity was also increased in the O-10a-KLF4 group compared O-10a hBM-MSCs (Fig. 5f).

### Implantation of miR-10a-overexpressed or KLF4-downregulated old hBM-MSCs into infarcted mouse hearts improved cardiac function after MI

**Fig. 6:**
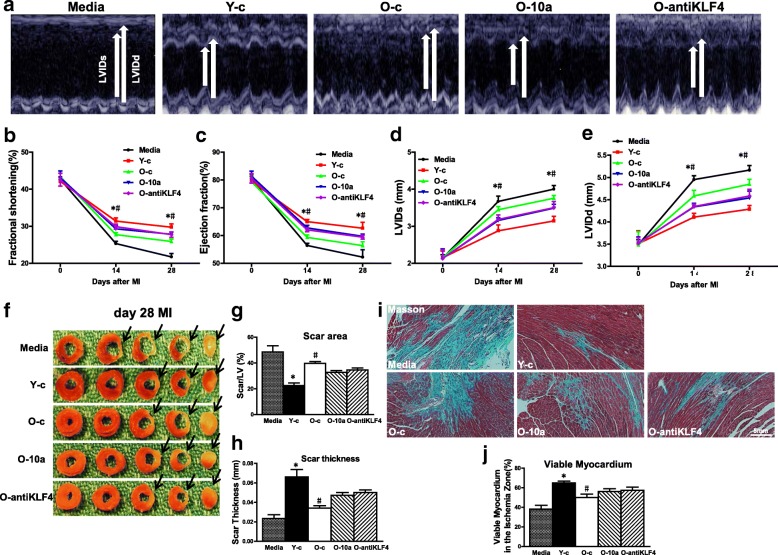
Implantation of miR-10a-overexpressed or KLF4-downregulated old hBM-MSCs into ischemic area of mouse hearts improved cardiac function after MI. miR-10a-overexpressed or KLF4-downregulated old hBM-MSCs (3 × 10^5^ cells/mouse) implanted into infarcted mouse hearts. Cardiac function determined by echocardiography in mice that received implantation of control medium (Media), control vector-transduced young hBM-MSCs (Y-c), control vector-transduced old hBM-MSCs (O-c), miR-10a-overexpressed old hBM-MSCs (O-10a), or KLF4-inhibited old hBM-MSCs (O-antiKLF4) into border region immediately following MI. Cardiac function measured by echocardiography at baseline (before MI, 0 day), 14 and 28 days after MI in all groups. **a** Representative M-mode echocardiographic images. **b** Fractional shortening. **c** Ejection fraction. **d** Left ventricular internal end-systolic dimension (LVIDs). **e** Left ventricular internal end-diastolic dimension (LVIDd). **f** Each panel shows one representative whole sectioned heart (from base to apex) at 28 days after MI from five individual groups (Media, Y-c, O-c, O-10a, or O-antiKLF4) to show scar areas (arrows) (**g**) and scar size thickness (**h**). **i** Representative images of Masson Trichrome’s staining at 28 days after MI. **j** Viable myocardium (identified as red with Trichrome’s staining) in ischemia zone quantified and expressed as percentage of total infarct area. *n* = 6/group. Mean ± SD. *P < 0.05, Y-c vs other groups; #*P* < 0.05, O-c vs other groups. LV left ventricle, MI myocardial infarction

To evaluate whether modifying miR-10a or KLF4 levels in O hBM-MSCs can maximize the beneficial effects of stem cell therapy, miR-10a-overexpressed or KLF4-downregulated old hBM-MSCs were implanted into infarcted mouse hearts. Cardiac function was determined by echocardiography in mice that received implantation of control medium (Media), control vector-transduced young hBM-MSCs (Y-c), control vector-transduced old hBM-MSCs (O-c), miR-10a-overexpressed old hBM-MSCs (O-10a), or KLF4-inhibited old hBM-MSCs (O-antiKLF4) into the border region immediately following MI. Cardiac function was evaluated at baseline (before MI) and at 14 and 28 days after MI. Fig. 6a shows representative M-mode echocardiographic images taken 28 days post MI in the four experimental groups. After MI, there was a significant decrease in fractional shortening (FS; Fig. 6b) and ejection fraction (EF; Fig. 6c) and an increase in left ventricular internal end-systolic dimension (LVIDs; Fig. 6d) and left ventricular internal end-diastolic dimension (LVIDd; Fig. 6e) in the O-c group compared with the Y-c group. However, there was an improvement in all of these parameters in the O-10a and O-antiKLF4 groups when compared with the O-c group (Fig. 6a–e).

Similarly, the infarct size at 28 days post MI was significantly larger in the O-c group than the Y-c group (Fig. 6f, g). On the other hand, scar thickness was significantly lower in the O-c group than the Y-c group (Fig. 6h). However, the infarct size was smaller and the scar thickness was greater in the O-10a and O-antiKLF4 groups when compared with the O-c group (Fig. 6f–h). More viable myocardium was found by Trichrome’s staining in the Y-c group than in the O-c group. However, there was more viable myocardium in the O-10a and O-antiKLF4 groups when compared with the O-c group (Fig. 6i, j). All of these data clearly demonstrated that modifying the miR-10a or KLF4 level in O hBM-MSCs enhanced the beneficial effects of stem cell therapy and further improved cardiac function.

### miR-10a upregulation or KLF4 downregulation increased old hBM-MSC survival and decreased apoptosis by activating AKT

**Fig. 7:**
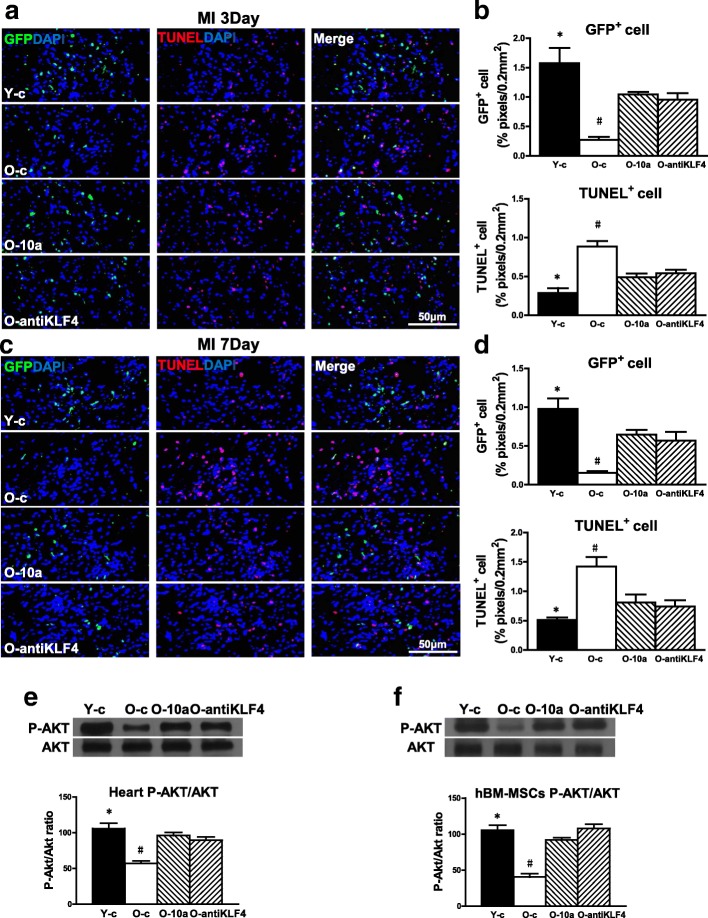
miR-10a overexpression or KLF4 downregulation increased old hBM-MSC survival and decreased apoptosis by activating AKT. miR-10a-overexpressed or KLF4-downregulated old hBM-MSCs (3 × 10^5^ cells/mouse) implanted into infarcted mouse hearts. Cell survival and biochemical changes determined in mice that received implantation of control vector-transduced young hBM-MSCs (Y-c), control vector-transduced old hBM-MSCs (O-c), miR-10a-overexpressed old hBM-MSCs (O-10a), or KLF4-inhibited old hBM-MSCs (O-antiKLF4) into border region immediately following MI. Survival of implanted cells detected by green fluorescent protein (GFP) expression carried by lentiviral vector in border region of infarcted mouse hearts and cell apoptosis assayed by TUNEL staining at 3 days (**a, b**) and 7 days (**c, d**) post MI. **e** Expression of total AKT and phospho-(ser473)-AKT detected in border region of infarcted mouse hearts tissue. **f** Expression of phosphor-(ser473)-AKT also detected in hBM-MSCs. *n* = 6/group. Mean ± SD. *P < 0.05, Y-c vs other groups; #*P* < 0.05, O-c vs other groups. DAPI 4′,6-diamidino-2-phenylindole, hBM-MSC human mesenchymal stem cell, KLF4 Krüpple-like factor 4, MI myocardial infarction, TUNEL terminal deoxynucleotidyl transferase dUTP nick end labeling

To evaluate the antiapoptotic effect of miR-10a in vivo, the survival of implanted cells was detected by lentiviral-mediated GFP expression in the border region of the mouse hearts at 3 days (Fig. 7a, b) and 7 days (Fig. 7c, d) post MI. Cell apoptosis was confirmed by TUNEL staining. There was highest cell survival and least apoptosis in the group receiving Y hBM-MSCs (Y-c). However, overexpression of miR-10a (O-10a) or inhibition of KLF4 expression (O-antiKLF4) increased cell survival but decreased cell apoptosis when compared with the group receiving O hBM-MSCs (O-c). To seek possible downstream mediators, western blot analyses were performed to examine the activation of AKT. As shown in Fig. 7e, although total AKT expression was similar for all the groups, phospho-Akt expression was highest in the Y-c group and was increased in the O-10a and O-antiKLF4 groups compared with the O-c group. The same pattern was found in the in-vitro cultured hBM-MSCs. This showed that total AKT expression was similar for hBM-MSCs from all of the conditions while phospho-AKT was highest in Y hBM-MSCs and was increased in the O-10a and O-antiKLF4 groups compared with O hBM-MSCs (Fig. 7f). To build up the direct link between the activation of Akt and increased cell survival, we used an inhibitor (Akt Inhibitor VI) to inhibit the phosphorylation (activation) of Akt in the miR-10a-overexpressed old hBM-MSCs (O-10a-P-AKT Inh; Additional file 9: Figure S8A). After culture for 72 h under hypoxia conditions, apoptosis was detected with the TUNEL assay (Additional file 9: Figure S8B) and cell survival was detected by CCK-8 assay in control vector-transduced (O-c), miR-10a-overexpressed (O-10a), and O-10a-P-AKT Inh old hBM-MSCs. We found that cell apoptosis was increased and cell survival was decreased in the O-10a-P-AKT Inh group compared to the O-10a group. We believe these results showed that miR-10a, through activating Akt, increased cell survival whereas inactivation of Akt reversed this effect. Collectively, these data revealed that miR-10a, through activation of AKT, increased hBM-MSC survival, thus improving cardiac function.

### miR-10a-overexpressed or KLF4-downregulated hBM-MSCs increased angiogenesis in infarcted mouse hearts

**Fig. 8:**
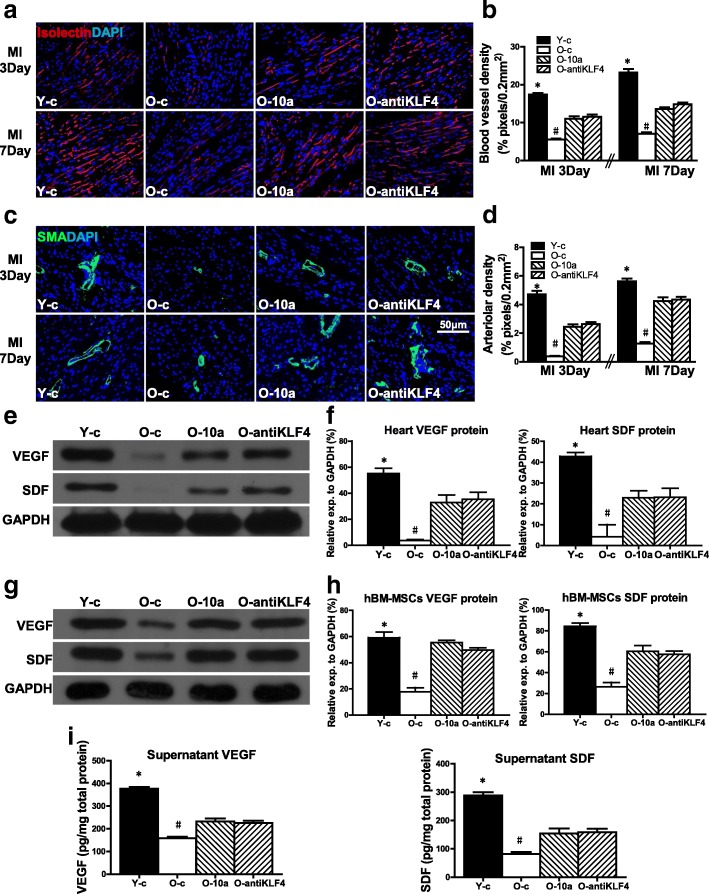
miR-10a-upregulated or KLF4-downregulated hBM-MSCs increased mice heart angiogenesis after MI. miR-10a-overexpressed or KLF4-downregulated old hBM-MSCs (3 × 10^5^ cells/mouse) implanted into infarcted mouse hearts. Angiogenesis and expression of angiogenic factors determined in mice that received implantation of control vector-transduced young hBM-MSCs (Y-c), control vector-transduced old hBM-MSCs (O-c), miR-10a-overexpressed old hBM-MSCs (O-10a), or KLF4-inhibited old hBM-MSCs (O-antiKLF4) into border region immediately following MI. Capillary and arteriole densities quantified by isolectin stain (**a, b**) and α-smooth muscle actin (α-SMA) stain respectively at 3 and 7 days (**c, d**) post MI. **e, f** VEGF and SDF protein expression evaluated by western blot analysis from border region of infarcted mouse hearts implanted with Y-c, O-c, O-10a, and O-antiKLF4 hBM-MSCs respectively. **g, h** VEGF and SDF protein expression evaluated by western blot analysis in Y-c, O-c, O-10a, and O-antiKLF4 hBM-MSCs. **i** VEGF and SDF protein in cell culture supernatant of Y-c, O-c, O-10a, and O-antiKLF4 hBM-MSCs assayed by ELISA. *n* = 6/group. Mean ± SD. *P < 0.05, Y-c vs other groups; #*P* < 0.05, O-c vs other groups. DAPI 4′,6-diamidino-2-phenylindole, hBM-MSC human mesenchymal stem cell, KLF4 Krüpple-like factor 4, MI myocardial infarction, SDF stromal cell-derived factor, VEGF vascular endothelial growth factor

To determine whether angiogenesis is also affected by regulating the miR-10a or KLF4 level in O hBM-MSCs, capillary and arteriole densities were quantified by isolectin stain (Fig. 8a, b) and α-smooth muscle actin (α-SMA) stain (Fig. 8c, d) respectively in all four experimental groups at 3 and 7 days post MI. More capillaries and arteriole were formed in the group receiving Y hBM-MSCs (Y-c) compared to the group receiving O hBM-MSCs (O-c). However, overexpression of miR-10a (O-10a) or inhibition of KLF4 (O-antiKLF4) increased capillary and arteriole formation when compared with the group receiving O hBM-MSC implantation only. Furthermore, the expression of angiogenic factors, VEGF and SDF mRNA (Additional file 10: Figure S9A), and protein (Fig. 8e, f) was decreased in the heart tissue from the border region of mice in the O hBM-MSC group (O-c) compared to the Y hBM-MSC group (Y-c). However, overexpression of miR-10a (O-10a) or inhibition of KLF4 e (O-antiKLF4) increased VEGF and SDF mRNA and protein expression when compared with the O-c group (Fig. 8e, f). The same trend was found in the in-vitro cultured hBM-MSCs. The VEGF and SDF mRNA (Additional file 10: Figure S9B) and protein (Fig. 8g, h) expression was decreased in O hBM-MSCs (O-c) compared with Y hBM-MSCs (Y-c). However, overexpression of miR-10a (O-10a) or inhibition of KLF4 (O-antiKLF4) increased VEGF and SDF mRNA (Additional file 10: Figure S9B) and protein expression compared with that in O hBM-MSCs (Fig. 8g, h). The secretion of VEGF and SDF in the cell culture supernatant was also increased in the O-10a and O-antiKLF4 groups compared with O hBM-MSCs (Fig. 8i).

## Discussion

The present study demonstrated that restoring the miR-10a level in O hBM-MSCs increased cell survival and decreased apoptosis under in-vitro hypoxia and in-vivo ischemic conditions by repressing the expression of KLF4. Accordingly, restoration of the miR-10a level in O hBM-MSCs increased the antiapoptotic-related protein expression and decreased the proapoptosis-related protein expression. Furthermore, inhibition of miR-10a target gene KLF4 also increased both cell survival and the antiapoptotic-related protein expression while it decreased cellular apoptosis and the proapoptosis-related protein expression. The molecular mechanism study revealed that transplantation of miR-10a-overexpressed or KLF4-downregulated O hBM-MSCs activated AKT in infarcted mouse hearts, which led to increased survival of implanted cells. Furthermore, transplantation of miR-10a or anti-KLF4-pretreated O hBM-MSCs increased the expression and secretion of angiogenic factors VEGF and SDF, which increased angiogenesis in infarcted mouse hearts. All of these effects of miR-10a ultimately led to enhanced efficacy of stem cell therapy and the improvement of cardiac function after MI.

BM-MSCs are an optimal cell type for therapeutic approaches for various pathological conditions including cardiovascular disease since the cells can be easily isolated and expanded in vitro [20]. However, the beneficial effects of BM-MSC therapy are restricted by the low survival rate and decreased proliferative capacity of the aged cells after cell transplantation [21]. The ischemic environment of the infarcted heart triggers a strong inflammatory and oxidative stress reaction and overproduction of factors related to apoptosis, which all lead to increased BM-MSC apoptosis and decreased cell survival [22]. Various attempts have been made to improve BM-MSC survival under hypoxic and ischemic conditions [23]. Here, we have shown that restoring miR-10a in aged hBM-MSCs increased cell survival and decreased apoptosis when transplanted into the ischemic mouse hearts.

MicroRNAs play an important role in cell proliferation, differentiation, and apoptosis [8]. Upregulation of specific miRNAs increased BM-MSC survival, making them more effective to repair the infarct damage [24, 25]. miR-10a has also been reported as a prosurvival factor. Previous studies have found that expression of miR10a/10b was controlled by TWIST-1 and via the NF-κB and P53 axis, to control TNF-α-induced (and stroma-dependent) apoptosis in clonal myeloid cells. Therefore, the TWIST-1/miR10/p53 axis can serve as a potential new target for therapeutic interventions in advanced myelodysplastic syndromes [13]. On the other hand, knockdown of miR-10a in the NPM1 mutated cell line OCI-AML3 decreased cellular survival and clonogenic growth [14]. miR-10a is also reported to regulate cell apoptosis in the human cumulus–oocytes complex [26]. In addition, Bim, the proapoptotic factor, is directly targeted by miR-10a, resulting in repressing Casp9 which is a crucial factor in the apoptotic pathway [27]. miR-10a delivered by exosomes sustains the number of chemotherapy-damaged granulosa cells and reduces the number of chemotherapy-damaged apoptotic granulosa cells following nitrogen mustard treatment for 24–48 h [28]. All of these findings support the notion that miR-10a is a prosurvival factor, which counteracts the apoptotic signals. Consistent with previous data, we also found that upregulation of miR-10a in aged hBM-MSCs decreased hypoxia-induced apoptosis and increased cell survival. To the contrary, downregulation of miR-10a in aged hBM-MSCs increased hypoxia-induced apoptosis and decreased cell survival. Implantation of miR-10a-overexpressed old hBM-MSCs into the ischemic area of mouse hearts improved cell survival and cardiac function after MI.

In our previous paper, using a luciferase reporter assay, we reported that KLF4 is a direct target of miR-10a. Also, miR-10a decreased cell senescence in aged hBM-MSCs by repressing KLF4 [7]. KLF4 can function as both a repressor and an activator of transcription factors related to cell cycle regulation, apoptosis, and differentiation. The expression of KLF4 can be increased by DNA damage, serum deprivation, and contact inhibition [29]. Recent research suggests KLF4 as a proapoptotic factor. A combination of KLF4 plasmid and apigenin treatment increases apoptosis in the human malignant neuroblastoma SK-N-DZ and IMR-32 cell lines compared with control vector or single treatment [15]. After a full-length complementary DNA or an antisense oligonucleotide of KLF4 was transfected into a human immortalized myelogenous leukemia line (K562 cells), cell growth was decreased and cell apoptosis was increased [16]. Epigenetic inhibition of KLF4 in B-cell lymphomas and particularly in classic Hodgkin lymphoma cases increases lymphoma survival and decreases apoptosis [17]. Research in resveratrol has found that KLF4 and early growth response-1 expression was increased by resveratrol, resulting in transcription factor 3 activation and cellular apoptosis [30]. KLF4 expression is increased in murine astrocytes when exposed to X-ray radiation, resulting in more double-strand DNA breaking, and at last cell apoptosis [31]. The apoptosis rate of SK-BR-3 breast cancer cells is increased and cell tumorigenicity is decreased by upregulation of KLF4 [32]. However, the role of KLF4 on hBM-MSC apoptosis still remains unclear. In this study, we found that downregulation of KLF4 in aged hBM-MSCs decreased hypoxia-induced apoptosis and increased cell survival. To the contrary, upregulation of KLF4 in aged hBM-MSCs increased hypoxia-induced apoptosis and decreased cell survival. Accordingly, upregulation of KLF4 resulted in decreased expression of the antiapoptotic proteins BCL2 and MCL1 but increased the expression of proapoptotic proteins BAX and PUMA, and caused activation of caspase-3, leading to completion of the apoptotic machinery. The implantation of KLF4-downregulated old hBM-MSCs into the ischemic area of mouse hearts improved cell survival and cardiac function after MI. We believed that KLF4 plays a proapoptotic role in aged hBM-MSCs.

AKT is a key factor involved in cell survival, proliferation, and metabolism [33]. Our data show that miR-10a overexpression or KLF4 downregulation activated AKT both in aged hBM-MSCs and in ischemic mouse hearts. We believe the antiapoptotic effect of miR-10a may be mediated through activation of AKT. In addition, we found that miR-10a overexpression or KLF4 downregulation increased VEGF and SDF expression and secretion. VEGF is a key factor for angiogenesis [34]. It has been reported that VEGF inhibits posthypoxic MSC death and increases MSC survival and regeneration in ischemic conditions [35, 36]. SDF also confers increased vasculogenesis and angiogenesis [37]. Our data suggested that miR-10a increased the survival of aged hBM-MSCs which in turn secreted more VEGF and SDF to stimulate angiogenesis in the infarcted hearts. Improved cell survival and enhanced angiogenesis acted synergistically to preserve heart function after MI.

## Conclusions

Our study demonstrated that miR-10a decreased aged hBM-MSC apoptosis and increased cell survival through suppression of KLF4. Transplantation of miR-10a-overexpressed or KLF4-downregulated old hBM-MSCs activated AKT and stimulated angiogenesis in the ischemic hearts, thereby improving cardiac function. miR-10a and KLF4-modified stem cells could be a potent vehicle to combine cell and gene therapies to improve heart function after injury.

## Additional files

Additional file 1: **Table S1.** qRT-PCR primer and miRNA RT primer sequences (DOCX 19 kb)

Additional file 2: **Figure S1.** Proapoptotic and antiapoptotic gene expression in old hBM-MSCs under hypoxia conditions. Quantification of mRNA expression of BAX and PUMA (proapoptotic), BCL2 and MCL1 (antiapoptotic) in Y and O hBM-MSCs. *n* = 6/group. Mean ± SD. *P < 0.05 (PDF 36 kb)

Additional file 3: **Figure S2.** Expression of miR-10a and KLF4 in old hBM-MSCs regulated by lentiviral vector. Lentiviral vector carrying miR-10a sequence used to transduce old hBM-MSCs (O-10a) and control vector-transduced old hBM-MSCs (O-c) served as control. miR-10a expression was significantly higher in O-10a than in control vector-transduced (O) hBM-MSCs **(A)**. Lentiviral vector carrying KLF4 siRNA sequence used to transduce old hBM-MSCs (O-anti-KLF4). KLF4 expression was significantly lower in O-antiKLF4 than in control vector-transduced O hBM-MSCs **(B)**. Lentiviral vector carrying anti-miR-10a sequence used to transduce old hBM-MSCs (O-anti10a). miR-10a expression was significantly lower in O-anti10a than in control vector-transduced O hBM-MSCs **(C)**. Lentiviral vector carrying KLF4 sequence used to transduce old hBM-MSCs (O-KLF4). KLF4 expression was significantly higher in O-KLF4 than in control vector-transduced O hBM-MSCs **(D)**. Lentivirus which carries KLF4 vector used to infect miR-10a-upregulated old hBM-MSCs (O-10a) to restore KLF4 expression (O-10a-KLF4). miR-10a-upregulated old hBM-MSCs (O-10a) also infected by the control lentivirus (O-10a-c). KLF4 expression restored in O-10a-KLF4 compared to O-10a-c hBM-MSCs **(E)**. *n* = 5/group. Mean ± SD. ^*^P < 0.05 O-c vs O-10a, O-anti-KLF4, O-anti-10a, and O-KLF4; ^#^*P* < 0.05 O-10a-KLF4 vs O-10a-c and O-10a (PDF 41 kb)

Additional file 4: **Figure S3.** Overexpression of miR-10a in old hBM-MSCs decreased apoptotic gene expression. Quantification of mRNA expression of BAX and PUMA (proapoptotic), BCL2 and MCL1 (antiapoptotic) in O and O-10a hBM-MSCs after culture for 72 h under hypoxia conditions. *n* = 6/group. Mean ± SD. *P < 0.05 (PDF 37 kb)

Additional file 5: **Figure S4.** Overexpression of miR-10a in both young and old hBM-MSCs decreased hypoxia-induced apoptosis and increased cell survival. miR-10a transduced into young (Y-10a) and old (O-10a) hBM-MSCs by lentiviral vector. Control vector-transduced young hBM-MSCs (Y-c) and old hBM-MSCs (O-c) served as controls. Cells cultured for 72 h under hypoxia conditions. (**A**) Cell apoptosis assayed by TUNEL staining. Percentage of apoptotic cells (TUNEL^+^) quantified in Y-c, Y-10a, O-c, and O-10ahBM-MSCs. (**B**) Cell survival evaluated in Y-c, Y-10a, O-c, and O-10a hBM-MSCs. *n* = 6/group. Mean ± SD. *P < 0.05, Y-c vs Y-10a; #*P* < 0.05, O-c vs O-10a (PDF 94 kb)

Additional file 6: **Figure S5.** Downregulation of KLF4 in old hBM-MSCs decreased apoptotic gene expression. Quantification of mRNA expression of BAX and PUMA (proapoptotic), BCL2 and MCL1 (antiapoptotic) in O and O-antiKLF4 hBM-MSCs after culture for 72 h under hypoxia conditions. *n* = 6/group. Mean ± SD. *P < 0.05 (PDF 37 kb)

Additional file 7: **Figure S6.** Downregulation of miR-10a or overexpression of KLF4 in old hBM-MSCs increased apoptotic gene expression. Quantification of mRNA expression of BAX and PUMA (pr-apoptotic), BCL2 and MCL1 (antiapoptotic) in O, O-anti10a and O-KLF4 hBM-MSCs after culture for 72 h under hypoxia conditions. *n* = 6/group. Mean ± SD. *P < 0.05 (PDF 38 kb)

Additional file 8: **Figure S7.** Antiapoptotic effect of miR-10a attenuated by restoration of KLF4 quantified by RT-qPCR. Quantification of mRNA expression of BAX and PUMA (proapoptotic), BCL2 and MCL1 (antiapoptotic) in O-10a-c, O-10a-KLF4, and O-c hBM-MSCs after culture for 72 h under hypoxia conditions. *n* = 6/group. Mean ± SD. *P < 0.05 (PDF 57 kb)

Additional file 9: **Figure S8.** miR-10a overexpression increased old hBM-MSC survival and decreased apoptosis by activating AKT. Expression of Akt phosphorylation in miR-10a-overexpressed old hBM-MSCs (O-10a) inhibited by Akt Inhibitor VI during 72-h culture under hypoxia conditions. **(A)** Expression of phosphor-(ser473)-AKT detected in O-c, O-10a, and Akt Inhibitor VI added O-10a hBM-MSCs (O-10a-P-AKT Inh). **(B)** Cell apoptosis assayed by TUNEL staining. Percentage of apoptotic cells (TUNEL^+^) quantified in O-c, O-10a, and O-10a-P-AKT Inh hBM-MSCs. **(C)** Cell survival evaluated in O-c, O-10a, and O-10a-P-AKT Inh hBM-MSCs. *n* = 5/group. Mean ± SD. ^*^P < 0.05, O-10a-P-AKT Inh vs O-10a (PDF 184 kb)

Additional file 10: **Figure S9.** Expression of VEGF and SDF mRNA in mouse hearts after MI and hBM-MSCs. **(A)** Expression of VEGF and SDF mRNA determined in border region of infarcted mouse hearts that received implantation of control vector-transduced young hBM-MSCs (Y-c), control vector-transduced old hBM-MSCs (O-c), miR-10a-overexpressed old hBM-MSCs (O-10a), or KLF4-inhibited old hBM-MSCs (O-antiKLF4) following MI. **(B)** Expression of VEGF and SDF mRNA determined by RT-qPCR in Y-c, O-c, O-10a, or O-antiKLF4 hBM-MSCs after hypoxia for 72 h. *n* = 6/group. Mean ± SD. *P < 0.05, Y-c vs other groups; ^#^*P* < 0.05, O-c vs other groups (PDF 39 kb)

## Abbreviations

BM: Bone marrow
CCK8: Cell Counting Kit-8
EF: Ejection fraction
FS: Fractional shortening
hBM-MSC: Human mesenchymal stem cell
KLF4: Krüpple-like factor 4
LVIDd: Left ventricular internal end-diastolic dimension
LVIDs: Left ventricular internal end-systolic dimension
MI: Myocardial infarction
SMA: Smooth muscle actin
TUNEL: Terminal deoxynucleotidyl transferase dUTP nick end labeling
