# Supplementary material for: miR-10a rejuvenates aged human mesenchymal stem cells and improves heart function after myocardial infarction through KLF4
Source: Stem Cell Res Ther. 2018 May 30;9:151. doi: 10.1186/s13287-018-0895-0 (PMC5977543; doi:10.1186/s13287-018-0895-0)
Supplement: Supplementary file 1 — Table S1. qRT-PCR primer and miRNA RT primer sequences (DOCX 19 kb) [file 13287_2018_895_MOESM1_ESM.docx]

**Table S1. qRT-PCR primer and miRNA RT primer sequences.**

| **Name** | **Forward Primer** | **Reverse Primer** |
| --- | --- | --- |
| **BAX** | **GTTGTCGCCCTTTTCTACTTTG** | **CCTGTAATCCCAGCTCCTTG** |
| **BCL2** | **GTGGATGACTGAGTACCTGAAC** | **GCCAGGAGAAATCAAACAGAGG** |
| **PUMA** | **CGACCTCAACGCACAGTAC** | **CCTAATTGGGCTCCATCTCG** |
| **SDF** | **GCTTGCTGAATTGGAAGTGAATG** | **TCTTCACGGAAACAGGGTTC** |
| **VEGF** | **AGTCCAACATCACCATGCAG** | **TTCCCTTTCCTCGAACTGATTT** |
| **MCL1** | **AAGGACAAAACGGGACTGG** | **ATATGCCAAACCAGCTCCTAC** |
| **GAPDH** | **AACGGATTTGGTCGTATTG** | **GGAAGATGGTGATGGGATT** |
| **KLF4** | **CCGCTCCATTACCAAGAGCT** | **TGGTCAGTTCATCTGAGCGG** |
| **miR-10a** | **GGATACCCTGTAGATCCGAA** | **CAGTGCGTGTCGTGGAGT** |
| **miR-10a**  **RT primer** | **GTCGTATCCAGTGCGTCGTGGAGTCGGCAATTGCACTGGATACGA**  **CCACAAA** | |
| **mouse SDF** | **ATGGAACCGATCAGTGTGAG** | **GATGAAGTAGATGGTGGGCAG** |
| **mouse Vegf** | **GGCAGCTTGAGTTAAACGAAC** | **TGGTGACATGGTTAATCGGTC** |
| **mouse GAPDH** | **CTTTGTCAAGCTCATTTCCTGG** | **TCTTGCTCAGTGTCCTTGC** |
