# Supplementary figures and images for: miR-10a rejuvenates aged human mesenchymal stem cells and improves heart function after myocardial infarction through KLF4
Source: Stem Cell Res Ther. 2018 May 30;9:151. doi: 10.1186/s13287-018-0895-0 (PMC5977543; doi:10.1186/s13287-018-0895-0)

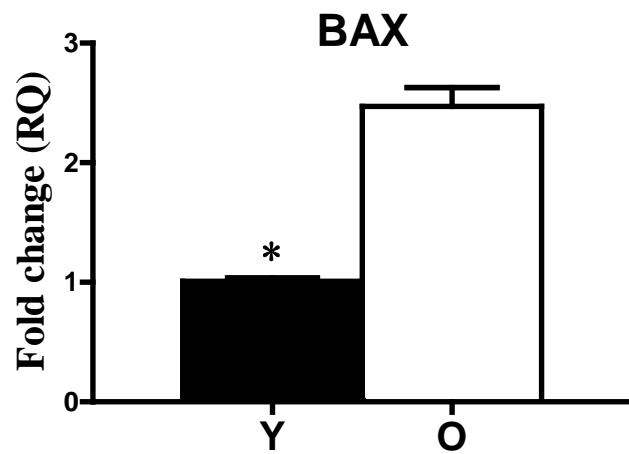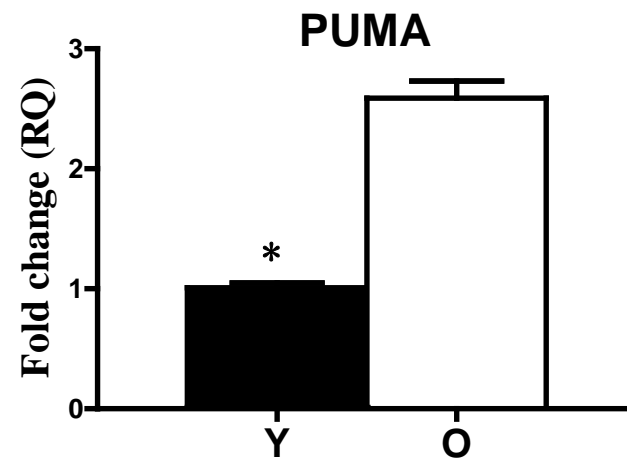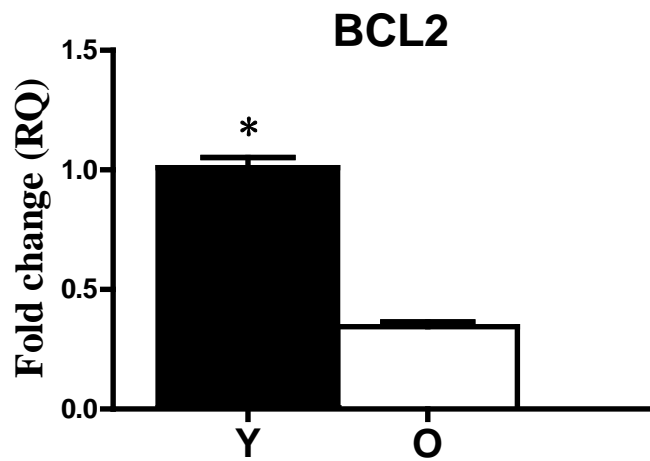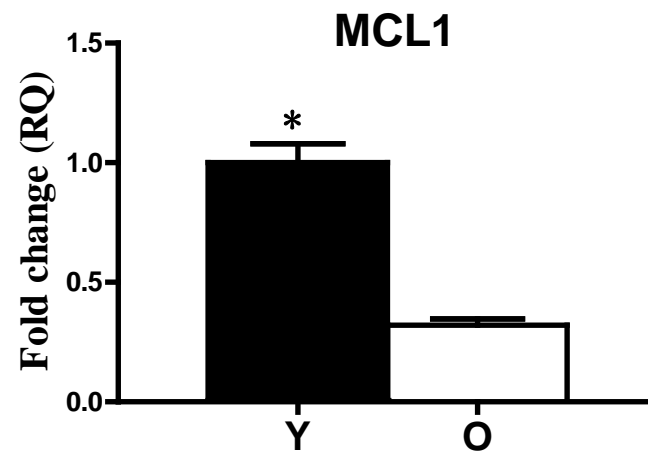

Supplement: Supplementary file 2 — Figure S1. Proapoptotic and antiapoptotic gene expression in old hBM-MSCs under hypoxia conditions. Quantification of mRNA expression of BAX and PUMA (proapoptotic), BCL2 and MCL1 (antiapoptotic) in Y and O hBM-MSCs. n = 6/group. Mean ± SD. *P < 0.05 (PDF 36 kb) [file 13287_2018_895_MOESM2_ESM.pdf]

**A** **MiR-10a expression**

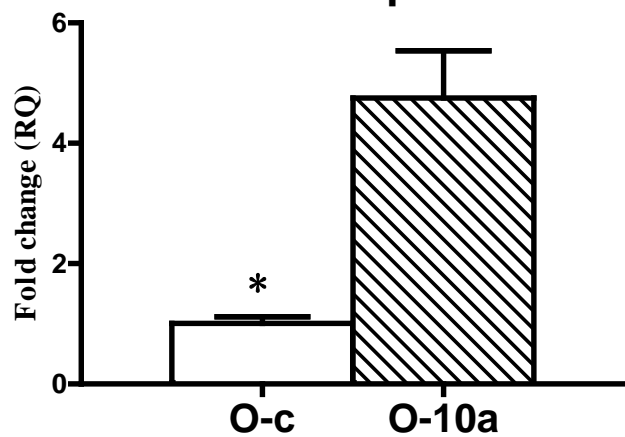

**B** **KLF4 expression**

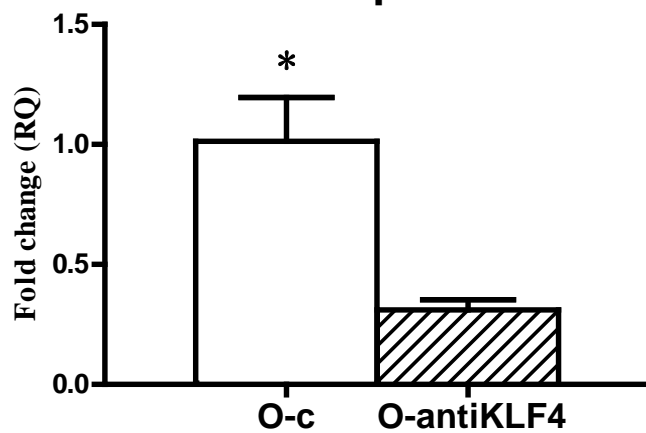

**C** **MiR-10a expression**

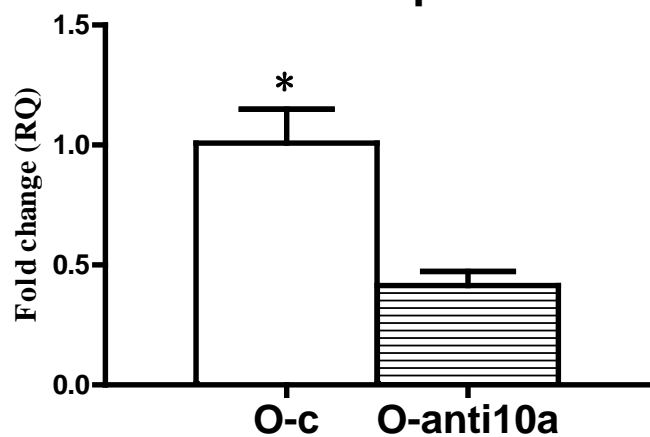

**D** **KLF4 expression**

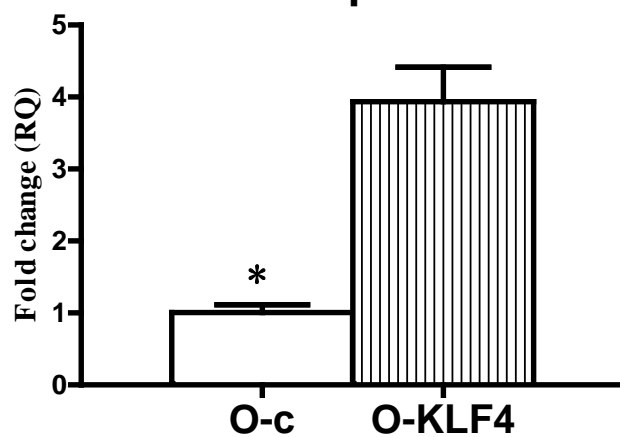

**E** **KLF4 expression**

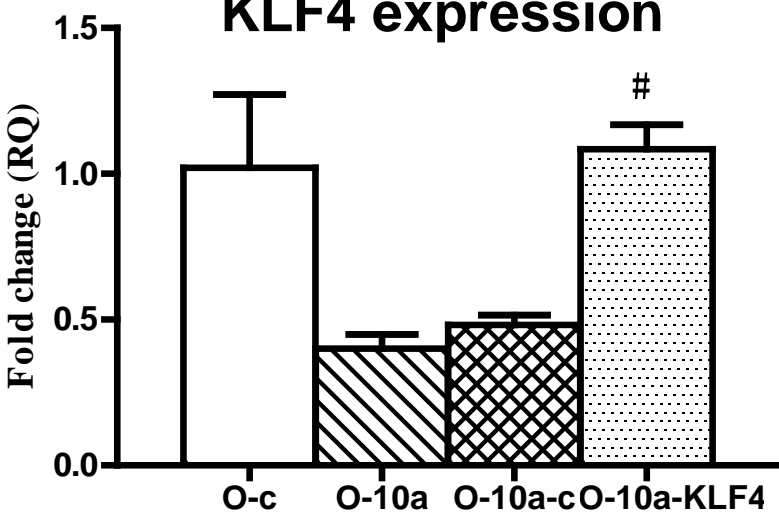

Supplement: Supplementary file 3 — Figure S2. Expression of miR-10a and KLF4 in old hBM-MSCs regulated by lentiviral vector. Lentiviral vector carrying miR-10a sequence used to transduce old hBM-MSCs (O-10a) and control vector-transduced old hBM-MSCs (O-c) served as control. miR-10a expression was significantly higher in O-10a than in control vector-transduced (O) hBM-MSCs (A). Lentiviral vector carrying KLF4 siRNA sequence used to transduce old hBM-MSCs (O-anti-KLF4). KLF4 expression was significantly lower in O-antiKLF4 than in control vector-transduced O hBM-MSCs (B). Lentiviral vector carrying anti-miR-10a sequence used to transduce old hBM-MSCs (O-anti10a). miR-10a expression was significantly lower in O-anti10a than in control vector-transduced O hBM-MSCs (C). Lentiviral vector carrying KLF4 sequence used to transduce old hBM-MSCs (O-KLF4). KLF4 expression was significantly higher in O-KLF4 than in control vector-transduced O hBM-MSCs (D). Lentivirus which carries KLF4 vector used to infect miR-10a-upregulated old hBM-MSCs (O-10a) to restore KLF4 expression (O-10a-KLF4). miR-10a-upregulated old hBM-MSCs (O-10a) also infected by the control lentivirus (O-10a-c). KLF4 expression restored in O-10a-KLF4 compared to O-10a-c hBM-MSCs (E). n = 5/group. Mean ± SD. *P < 0.05 O-c vs O-10a, O-anti-KLF4, O-anti-10a, and O-KLF4; #P < 0.05 O-10a-KLF4 vs O-10a-c and O-10a (PDF 41 kb) [file 13287_2018_895_MOESM3_ESM.pdf]

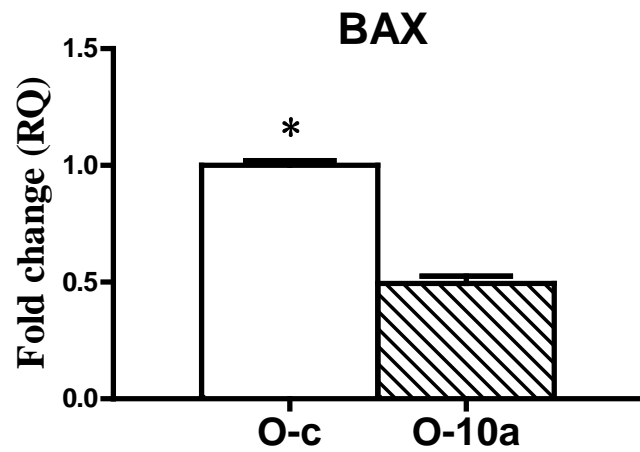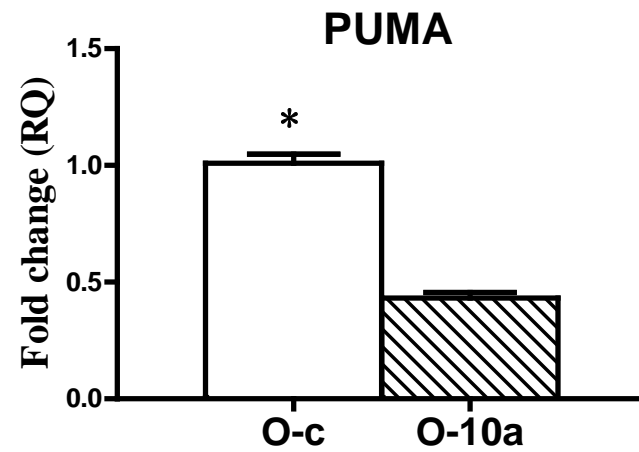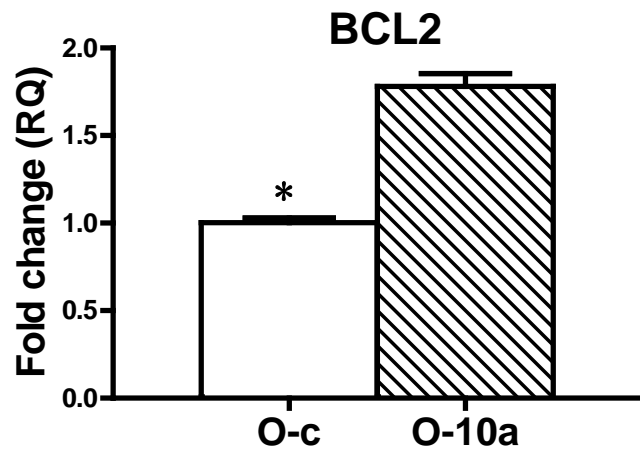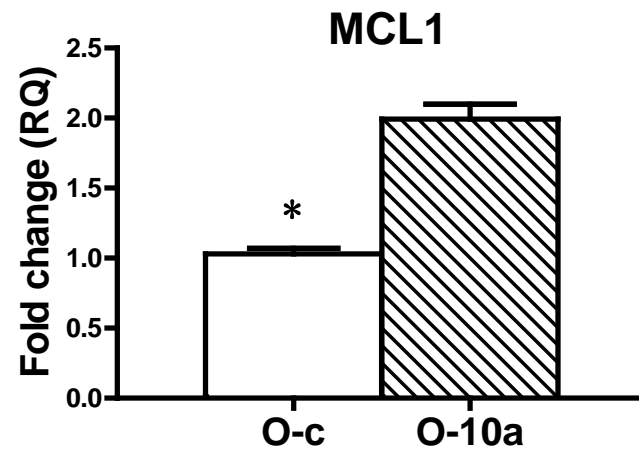

Supplement: Supplementary file 4 — Figure S3. Overexpression of miR-10a in old hBM-MSCs decreased apoptotic gene expression. Quantification of mRNA expression of BAX and PUMA (proapoptotic), BCL2 and MCL1 (antiapoptotic) in O and O-10a hBM-MSCs after culture for 72 h under hypoxia conditions. n = 6/group. Mean ± SD. *P < 0.05 (PDF 37 kb) [file 13287_2018_895_MOESM4_ESM.pdf]

**A**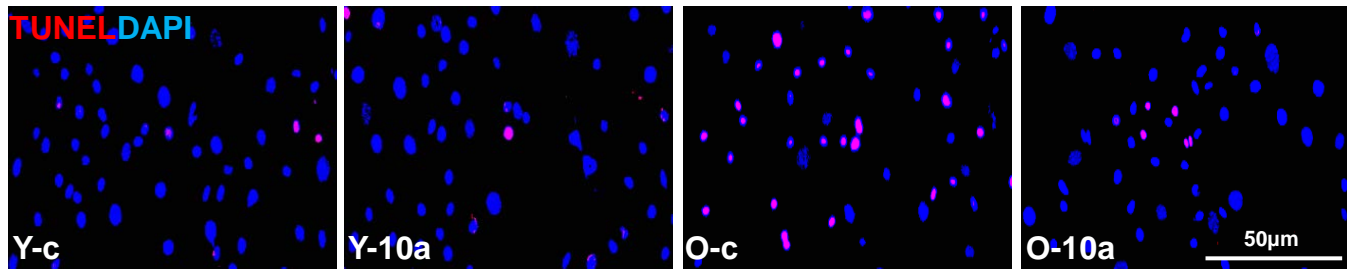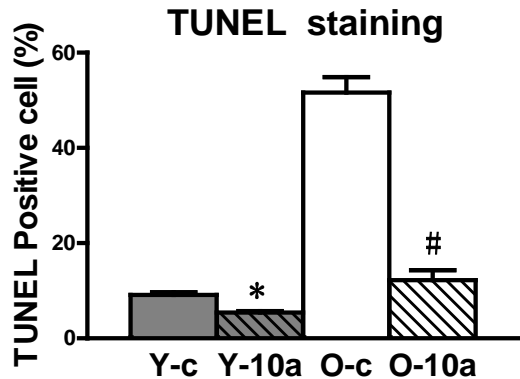**B**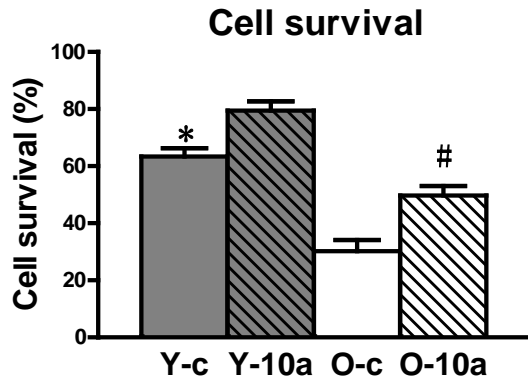

Supplement: Supplementary file 5 — Figure S4. Overexpression of miR-10a in both young and old hBM-MSCs decreased hypoxia-induced apoptosis and increased cell survival. miR-10a transduced into young (Y-10a) and old (O-10a) hBM-MSCs by lentiviral vector. Control vector-transduced young hBM-MSCs (Y-c) and old hBM-MSCs (O-c) served as controls. Cells cultured for 72 h under hypoxia conditions. (A) Cell apoptosis assayed by TUNEL staining. Percentage of apoptotic cells (TUNEL+) quantified in Y-c, Y-10a, O-c, and O-10ahBM-MSCs. (B) Cell survival evaluated in Y-c, Y-10a, O-c, and O-10a hBM-MSCs. n = 6/group. Mean ± SD. *P < 0.05, Y-c vs Y-10a; #P < 0.05, O-c vs O-10a (PDF 94 kb) [file 13287_2018_895_MOESM5_ESM.pdf]

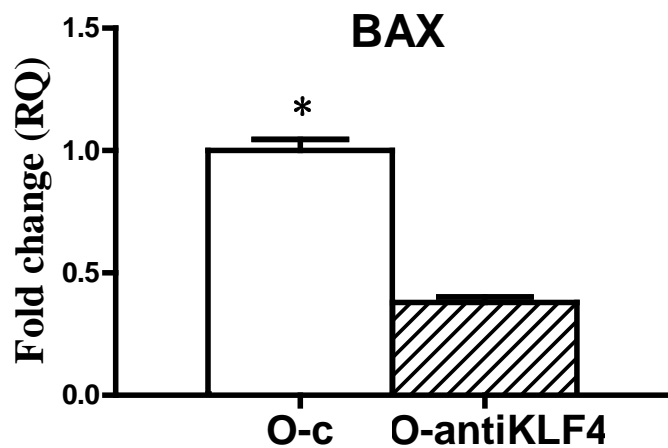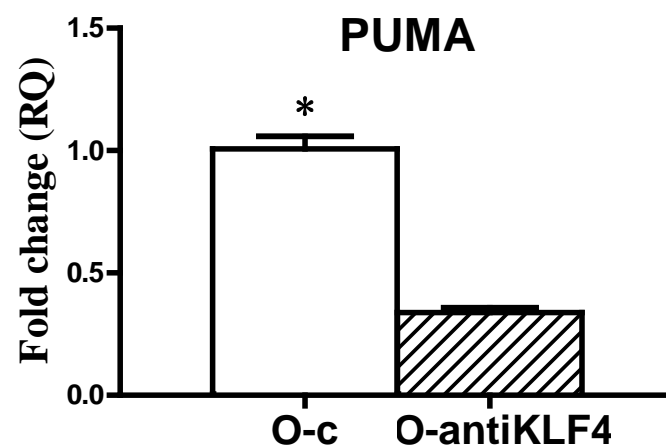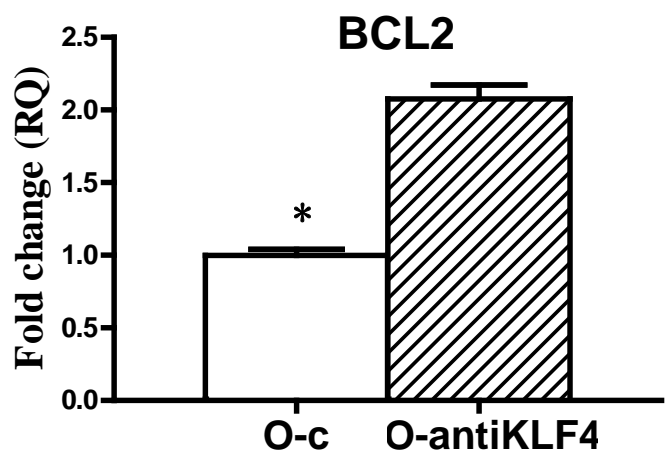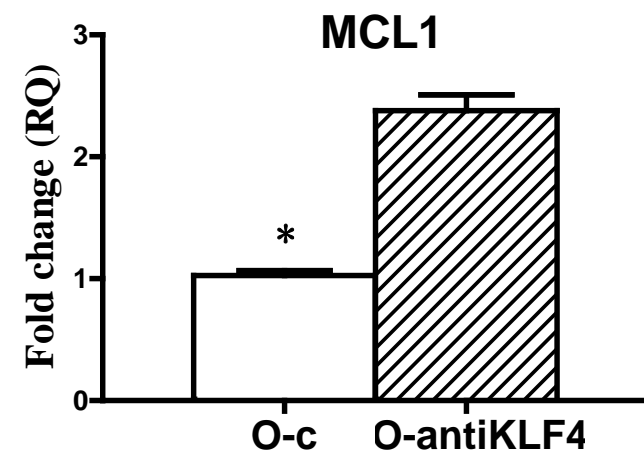

Supplement: Supplementary file 6 — Figure S5. Downregulation of KLF4 in old hBM-MSCs decreased apoptotic gene expression. Quantification of mRNA expression of BAX and PUMA (proapoptotic), BCL2 and MCL1 (antiapoptotic) in O and O-antiKLF4 hBM-MSCs after culture for 72 h under hypoxia conditions. n = 6/group. Mean ± SD. *P < 0.05 (PDF 37 kb) [file 13287_2018_895_MOESM6_ESM.pdf]

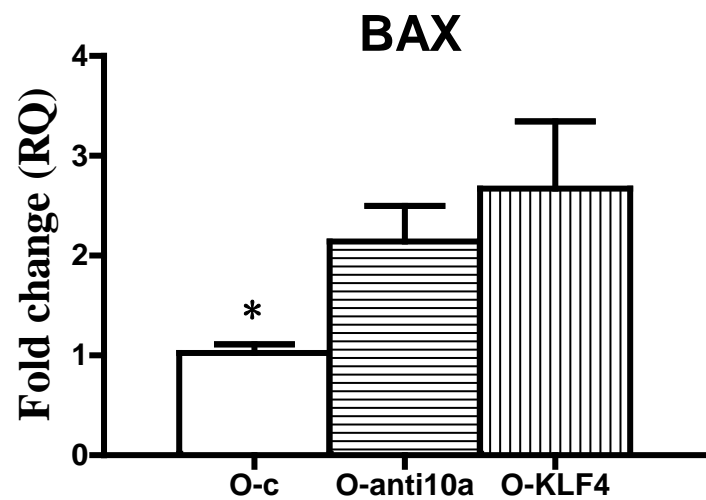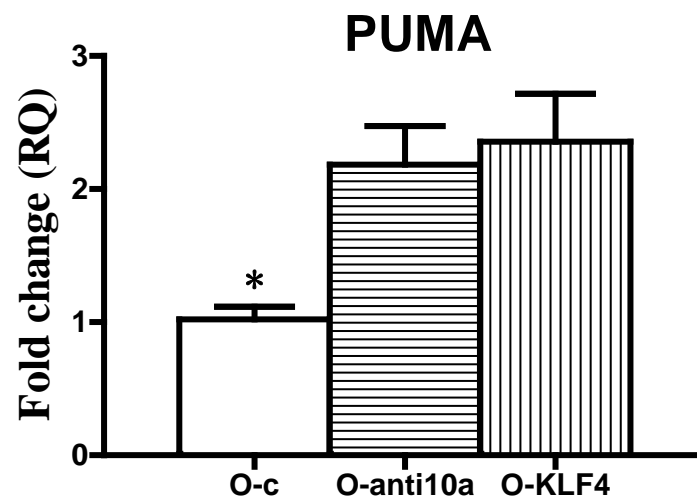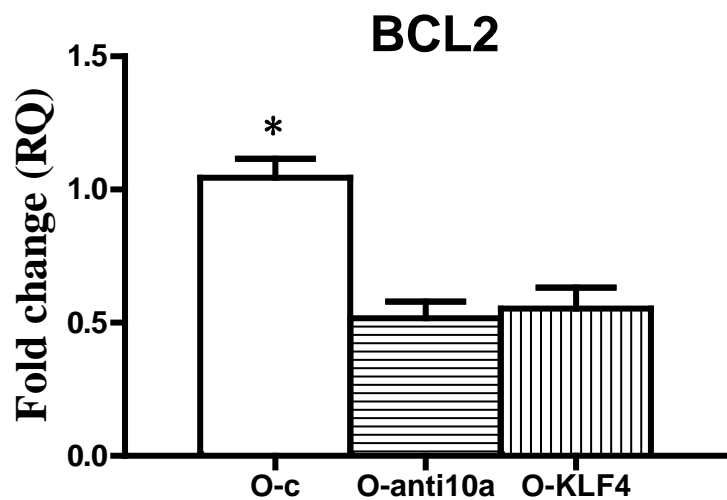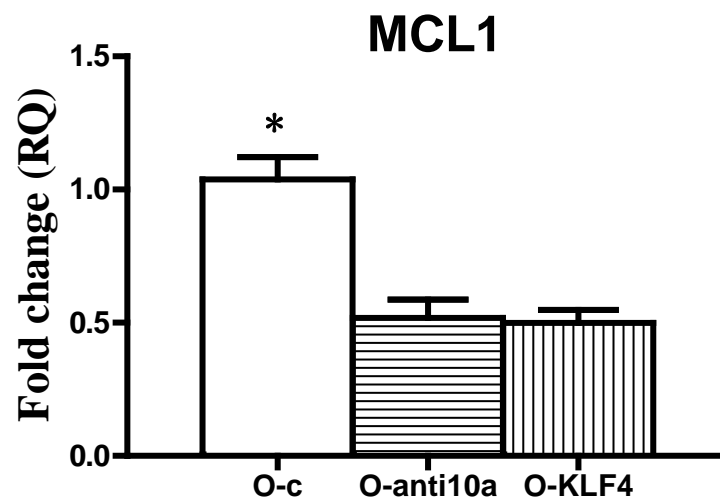

Supplement: Supplementary file 7 — Figure S6. Downregulation of miR-10a or overexpression of KLF4 in old hBM-MSCs increased apoptotic gene expression. Quantification of mRNA expression of BAX and PUMA (pr-apoptotic), BCL2 and MCL1 (antiapoptotic) in O, O-anti10a and O-KLF4 hBM-MSCs after culture for 72 h under hypoxia conditions. n = 6/group. Mean ± SD. *P < 0.05 (PDF 38 kb) [file 13287_2018_895_MOESM7_ESM.pdf]

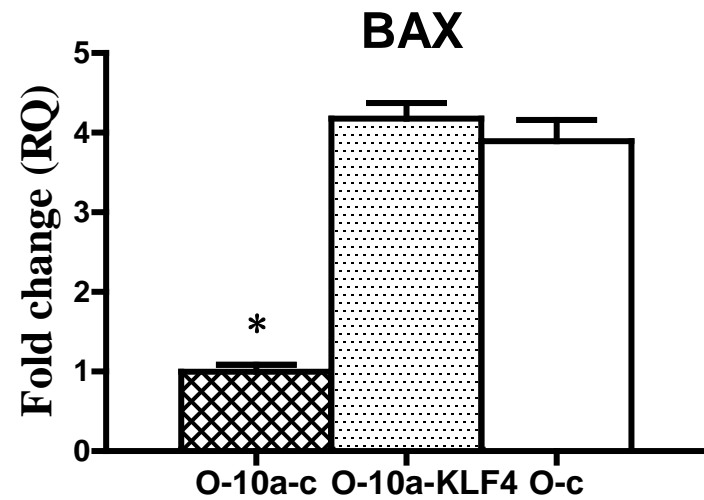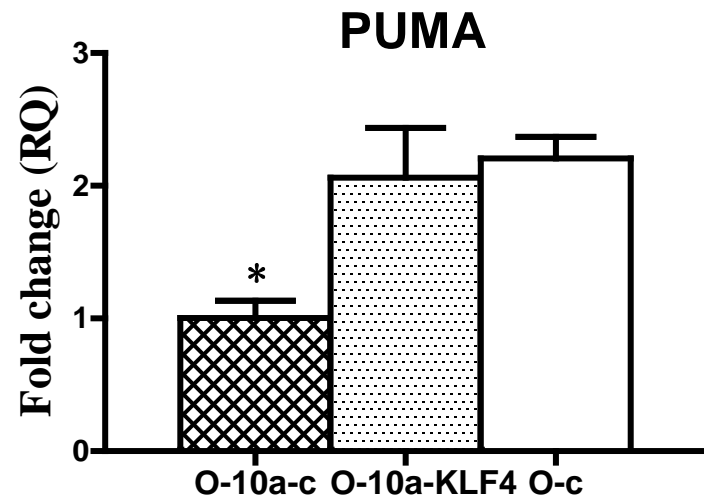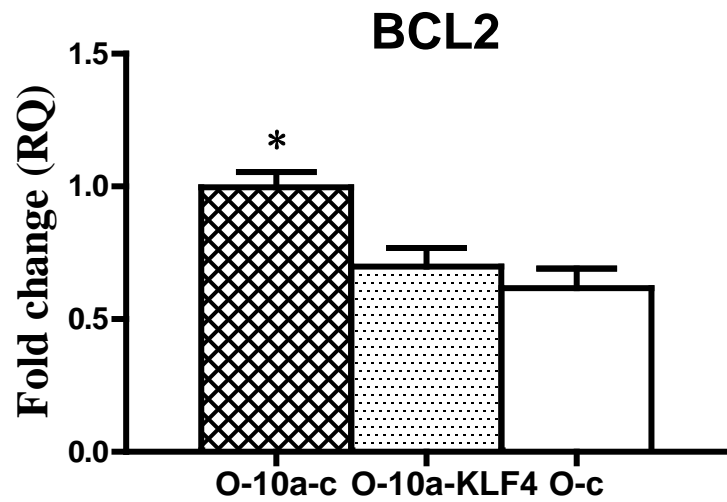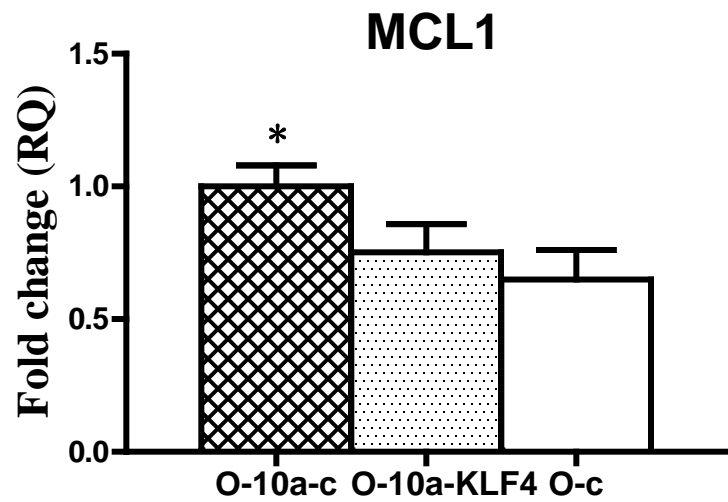

Supplement: Supplementary file 8 — Figure S7. Antiapoptotic effect of miR-10a attenuated by restoration of KLF4 quantified by RT-qPCR. Quantification of mRNA expression of BAX and PUMA (proapoptotic), BCL2 and MCL1 (antiapoptotic) in O-10a-c, O-10a-KLF4, and O-c hBM-MSCs after culture for 72 h under hypoxia conditions. n = 6/group. Mean ± SD. *P < 0.05 (PDF 57 kb) [file 13287_2018_895_MOESM8_ESM.pdf]

**A**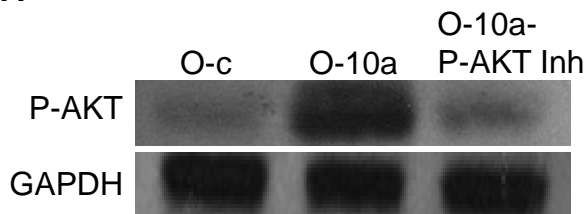**hBM-MSCs P-AKT**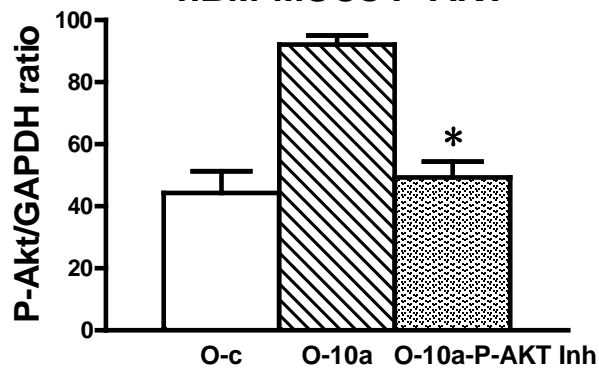**B**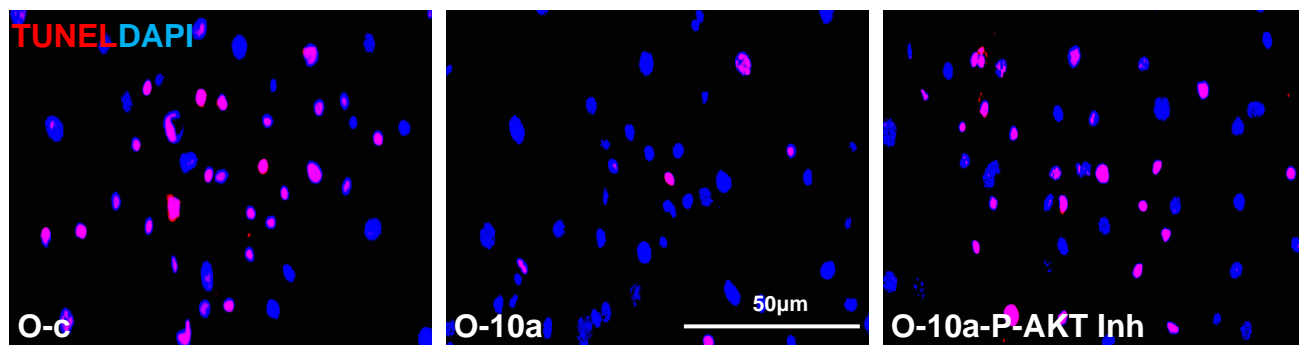**C**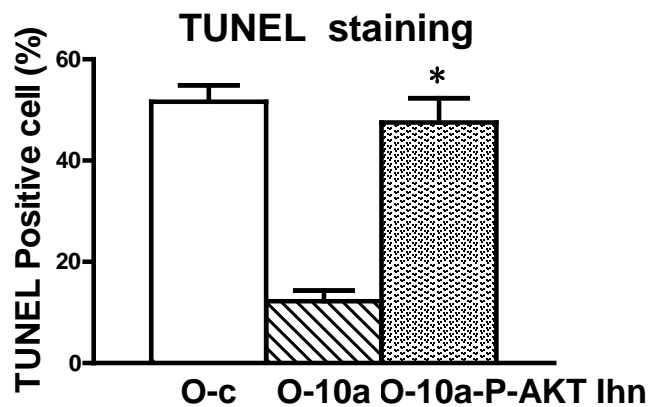**Cell survival**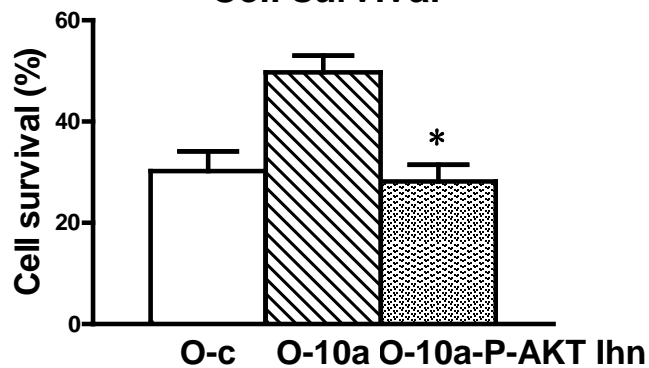

Supplement: Supplementary file 9 — Figure S8. miR-10a overexpression increased old hBM-MSC survival and decreased apoptosis by activating AKT. Expression of Akt phosphorylation in miR-10a-overexpressed old hBM-MSCs (O-10a) inhibited by Akt Inhibitor VI during 72-h culture under hypoxia conditions. (A) Expression of phosphor-(ser473)-AKT detected in O-c, O-10a, and Akt Inhibitor VI added O-10a hBM-MSCs (O-10a-P-AKT Inh). (B) Cell apoptosis assayed by TUNEL staining. Percentage of apoptotic cells (TUNEL+) quantified in O-c, O-10a, and O-10a-P-AKT Inh hBM-MSCs. (C) Cell survival evaluated in O-c, O-10a, and O-10a-P-AKT Inh hBM-MSCs. n = 5/group. Mean ± SD. *P < 0.05, O-10a-P-AKT Inh vs O-10a (PDF 184 kb) [file 13287_2018_895_MOESM9_ESM.pdf]

**A**

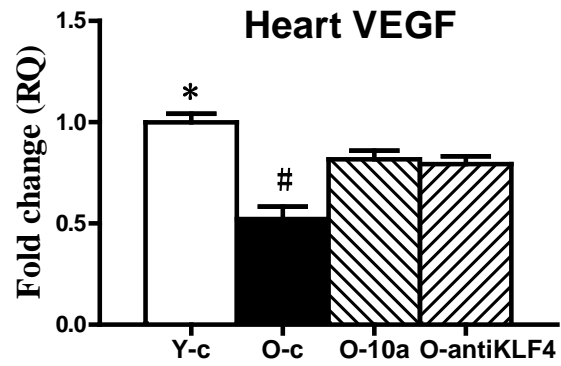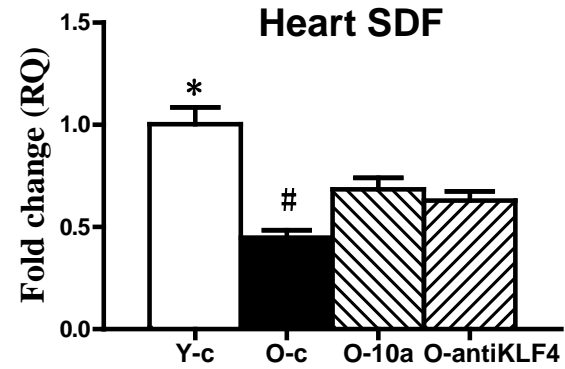

**B**

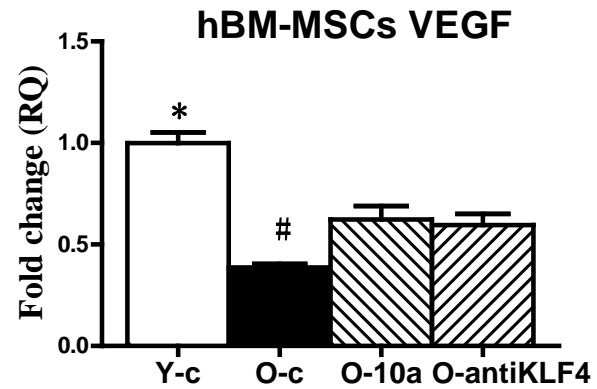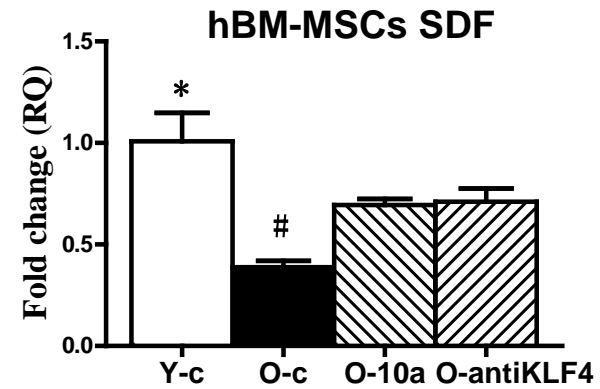

Supplement: Supplementary file 10 — Figure S9. Expression of VEGF and SDF mRNA in mouse hearts after MI and hBM-MSCs. (A) Expression of VEGF and SDF mRNA determined in border region of infarcted mouse hearts that received implantation of control vector-transduced young hBM-MSCs (Y-c), control vector-transduced old hBM-MSCs (O-c), miR-10a-overexpressed old hBM-MSCs (O-10a), or KLF4-inhibited old hBM-MSCs (O-antiKLF4) following MI. (B) Expression of VEGF and SDF mRNA determined by RT-qPCR in Y-c, O-c, O-10a, or O-antiKLF4 hBM-MSCs after hypoxia for 72 h. n = 6/group. Mean ± SD. *P < 0.05, Y-c vs other groups; #P < 0.05, O-c vs other groups (PDF 39 kb) [file 13287_2018_895_MOESM10_ESM.pdf]
